# Application of Nanostructured Carbon-Based Electrochemical (Bio)Sensors for Screening of Emerging Pharmaceutical Pollutants in Waters and Aquatic Species: A Review

**DOI:** 10.3390/nano10071268

**Published:** 2020-06-29

**Authors:** Álvaro Torrinha, Thiago M. B. F. Oliveira, Francisco W.P. Ribeiro, Adriana N. Correia, Pedro Lima-Neto, Simone Morais

**Affiliations:** 1 REQUIMTE-LAQV, Instituto Superior de Engenharia do Porto, Instituto Politécnico do Porto, Rua Dr. António Bernardino de Almeida, 431, 4249-015 Porto, Portugal; alvaro.torrinha@graq.isep.ipp.pt; 2 Centro de Ciência e Tecnologia, Universidade Federal do Cariri, Av. Tenente Raimundo Rocha, 1639, Cidade Universitária, 63048-080 Juazeiro do Norte, CE, Brazil; thiago.mielle@ufca.edu.br; 3 Instituto de Formação de Educadores, Universidade Federal do Cariri, Rua Olegário Emídio de Araújo, S/N, Centro, 63260-000 Brejo Santo - CE, Brazil; wirley.ribeiro@ufca.edu.br; 4 GELCORR, Departamento de Química Analítica e Físico-Química, Centro de Ciências, Universidade Federal do Ceará, Bloco 940, Campus do Pici, 60455-970 Fortaleza-CE, Brazil; adriana@ufc.br (A.N.C.); pln@ufc.br (P.L.-N.)

**Keywords:** sensors and biosensors, carbon nanomaterials, environment, aquatic fauna, waters

## Abstract

Pharmaceuticals, as a contaminant of emergent concern, are being released uncontrollably into the environment potentially causing hazardous effects to aquatic ecosystems and consequently to human health. In the absence of well-established monitoring programs, one can only imagine the full extent of this problem and so there is an urgent need for the development of extremely sensitive, portable, and low-cost devices to perform analysis. Carbon-based nanomaterials are the most used nanostructures in (bio)sensors construction attributed to their facile and well-characterized production methods, commercial availability, reduced cost, high chemical stability, and low toxicity. However, most importantly, their relatively good conductivity enabling appropriate electron transfer rates—as well as their high surface area yielding attachment and extraordinary loading capacity for biomolecules—have been relevant and desirable features, justifying the key role that they have been playing, and will continue to play, in electrochemical (bio)sensor development. The present review outlines the contribution of carbon nanomaterials (carbon nanotubes, graphene, fullerene, carbon nanofibers, carbon black, carbon nanopowder, biochar nanoparticles, and graphite oxide), used alone or combined with other (nano)materials, to the field of environmental (bio)sensing, and more specifically, to pharmaceutical pollutants analysis in waters and aquatic species. The main trends of this field of research are also addressed.

## 1. Introduction

The unintended presence of pharmaceuticals in the environment has been raising awareness from the scientific community and regulatory authorities given the possible adverse effects on aquatic ecosystems and human health. With the world population increasing, and predicted to reach 9.7 billion by 2050 [1] in conjugation with the increase of life expectancy, the pressure caused by pollutants and particularly pharmaceuticals on the environment is clearly expected also to rise. This seems to be avoidable or at least mitigated if preventive measures and efficient treatment procedures become implemented soon. For instance, information and knowledge acquired through efficient monitoring methods may have a crucial role in the environment preservation by contributing to establish regulation on maximum levels and effective measures against this problem.

The occurrence of pharmaceuticals in the environment are mainly due to technological limitations in wastewater treatment (WWT) related with anthropogenic activities [2,3,4]. Conventional wastewater treatments, still widely implemented as main processes, cannot efficiently remove pharmaceuticals from effluents [5,6,7]. Inefficiency of WWT in the total removal of pharmaceuticals is proved by different recent studies that have detected pharmaceuticals in the range of ng L^−1^ to μg L^−1^ in water samples collected nearby wastewater discharges or in effluents from medical care units and municipal treatment plants [8,9,10,11]. Other pathways of aquatic contamination are related with the application of veterinary drugs in aquaculture and agriculture [12,13,14,15].

Pharmaceuticals are designed to perform specific biological functions within an organism during a period until excretion. Their inherent physicochemical properties makes them to be, at some extent, persistent, liable to bioaccumulate in living tissues and toxic (designated as PBT substances) [3]. In this perspective, the OSPAR commission [16], which is dedicated to the protection and conservation of the North-East Atlantic Ocean and its resources, has identified about 25 pharmaceutical drugs and hormones (17α-ethinylestradiol, 17β-estradiol, chloroquine, chlorpromazine, closantel, clotrimazole, diethylstilbestrol, dimetacrine, estrone, flunarizine, fluoxetine, fluphenazine, mestranol, miconazole, midazolam, mitotane, niflumic acid, niclofolan, fluphenazine, pimozide, prochlorperazine, penfluridol, trifluoperazine, trifluperidol, timiperone) that could negatively affect marine ecosystems based on their PBT characteristics. However, it is surprising that most of these pharmaceuticals are not considered in pharmaceutical screening studies, probably explained by their low worldwide consumption.

Although not considered as persistent as other pollutants (such as organochlorine pesticides, polychlorinated biphenyls, and dioxins) [17,18,19], pharmaceuticals’ continuous use and subsequent discharge makes them ubiquitous in the environment and therefore termed as ‘pseudo-persistent’ compounds [3,4]. The bioavailability of pharmaceuticals makes them susceptible to ingestion and absorption by the surrounding fauna, as demonstrated by several studies focused on biota analyses [4,20,21,22], which is suggestive that bioaccumulation can occur. In a study conducted by Howard and Muir [23], about 92 pharmaceuticals were estimated to be potentially bioaccumulative from a database of 275 frequently found in the environment. Chronic exposure to pseudo persistent pharmaceuticals, even at trace levels, can have a significant impact on non-target organisms. The negative effects of endocrine disruptive compounds (EDC) on the reproductive characteristics and behavior of organisms’ aquatic fauna are well documented. Synthetic hormones such as 17α-ethinylestradiol or diethylstilbestrol are examples of potent EDC [24], that are included as well as other hormones in the EPA Contaminant Candidate List as priority for information and regulation measures [25]. Non-steroidal anti-inflammatories [26,27] and antidepressants [28] are other classes of drugs with evidence of disruptive capacity. Also the exposure to antibiotics that are continuously released through wastewater discharges or as veterinary drugs in aquaculture activities may affect natural microorganisms leading to bacterial resistance, posing at risk aquatic fauna and consequently human health [29]. Another concern on ecotoxicity is the synergetic effect that multiple drugs seem to exert in non-target organisms [30,31], however, information on possible effects of mixtures is still scarce and with unpredictable results [2,32].

**Scheme 1:**
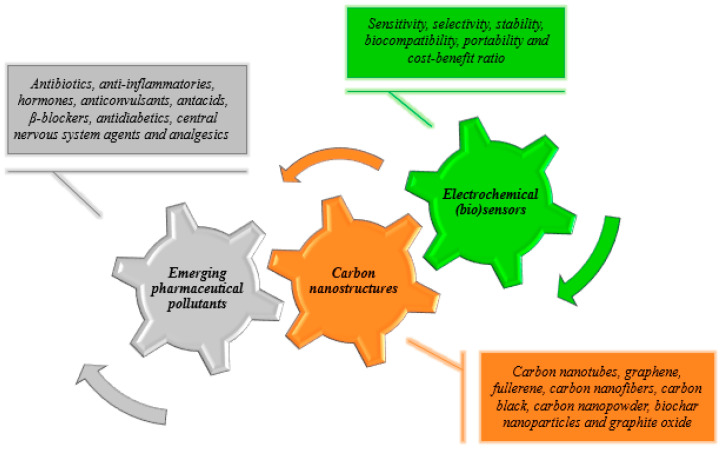
Characteristics and advantages of nanostructured carbon-based electrochemical (bio)sensors to quantify pharmaceuticals as emergent pollutants in different matrices.

Environmental analysis of pharmaceuticals has been predominantly performed in aqueous matrices, failing to give a more extended and comprehensive risk assessment [4]. Furthermore, these analyses have been mostly performed through hyphenated methods, conjugating separation, and detection. Chromatography and spectroscopic detection based on mass spectrometry or spectrophotometry have been widely applied as analytical techniques of choice since they enable multi-residue analysis with high selectivity and good sensitivity [33,34]. Yet, although being very reliable and efficient methods, they are also bulky and expensive techniques requiring highly specialized personnel for their operation. Thus, there is an excellent scope for the application of sensor technology comprising a range of different techniques including chemical sensors and biosensors. In pharmaceutical analysis, sensor technology is mostly based on electrochemical and optical (fluorescence, colorimetric, surface plasmon resonance, etc.) detection principles [35,36]. Particularly, electrochemical (bio)sensors constitute a versatile and viable option, meeting sustainable practices by using reduced sample volumes and reagents [37,38]. This technology relies on modified electrode surfaces for transduction of redox reactions. The signal generated by the transfer of electrons between the transducer and the analyte is amplified in the equipment and finally displayed [39,40]. The possibility of designing portable and simple (bio)sensor devices at lower costs enables in-situ applications, which is a major advantage and therefore a viable alternative to the more conventional chromatographic methods in respect to environmental analysis. Moreover, a competitive characteristic of electrochemical (bio)sensors is their potential to be miniaturized, with emphasis on the contribution of nanotechnology in this process. Nanostructuration based on carbon materials takes advantage of their unique properties enabling the construction of (bio)sensors with enhanced performance with high surface-to-volume ratio (Scheme 1). Since this type of (bio)sensor is based on the processing of an electrical signal, it seems evident the importance of conductive materials in the enhancement of that signal. Although carbon nanomaterials have lower conductivity compared to metals, they present a metallic or semiconductive behavior [41] (resistivities in the order of 10^−4^ Ω cm [42,43]) suitable to achieve high electron transfer rates. Furthermore, the easy processing of a relatively abundant chemical element (carbon) enables facile fabrication and commercial availability of carbon nanomaterials at acceptable cost. These are significant advantages over other competitive nanomaterials (essentially metallic nanoparticles), leading to their wide application in (bio)sensors technology [44,45,46]. However, the research available on this subject is still very limited, especially regarding biota analysis [35,36,47,48].

In the present study, a literature review is carried-out concerning the use of electrochemical (bio)sensors exclusively nanostructured with carbon-based materials (single and multi-walled carbon nanotubes, graphene, fullerene, carbon nanofibers, carbon black, carbon nanopowder, biochar nanoparticles, and graphite oxide) for emerging pharmaceutical pollutants detection in waters and aquatic species. Here, we give insight on the characteristics of the different carbon nanomaterials (and developed nanocomposites) used in the (bio)sensor assembly, addressing the achieved electroanalytical performance as comparative criteria between the described (bio)sensors. As far as we know, this is the first review exclusively focused on the application of electrochemical (bio)sensors for pharmaceuticals detection in the selected matrices. Moreover, this work clearly shows the high contribution of nanomaterials towards the development of extremely sensitive (bio)sensors.

A total of 108 pharmaceutical drugs were considered in the literature search for the review. The selective criterion was based in those frequently found in the environment accordingly to recent chromatographic studies dedicated to pharmaceuticals monitoring in aquatic fauna [22,49,50,51,52,53,54,55,56,57,58] and waters [8,9,10,11,59,60,61,62]. Additionally, the 25 pharmaceuticals included in the list of OSPAR Commission [16] as well as the top 20 most consumed in Portugal [63] and USA [64] were also taken in consideration.

## 2. Nanostructured Carbon-Based (Bio)Sensors for Pharmaceutical Pollutants

### 2.1. Graphene

Graphene is a single-crystal layer of *sp*^2^-bonded carbon atoms (honeycomb-like 2D arrangement), which represents the basic form to build all other carbon allotropes. (Bio)sensors based on this nanomaterial demonstrate an impressive performance to monitor the diffusion and persistence of pharmaceutical and their metabolites in diverse matrices [65,66,67] being therefore one of the most described nanomaterials in the present review with a total of 21 developed (bio)sensors (Table 1).

As expected, antibiotic pharmaceuticals were widely studied by graphene-based (bio)sensors due to their ubiquitous presence in the environment. In this sense, assuming possible contamination by antibiotics in Chinese breeding sites and subsequent deleterious effects on consumers’ health, Chen et al. [68] developed a fast and accurate electroanalytical method to assess the presence of ciprofloxacin in shrimp and sea cucumber cultures. The quantification was performed by differential pulse voltammetry (DPV), using a sensor based on graphene oxide (GO) dispersed in a hybrid matrix of sodium polyacrylate-chloropalladic acid. The authors achieved a wide linear range and sensitivity above those obtained with other electrochemical platforms structured with multi-walled carbon nanotubes (MWCNT), MgFe_2_O_4_-MWCNT, β-cyclodextrin/L-arginine, poly(alizarin red), and horseradish peroxidase. Silva and Cesarino [69] proposed a nanocomposite electrochemical sensor for sulfamethazine detection based on gold nanoparticles (AuNPs) and reduced graphene oxide (rGO) immobilized on glassy carbon electrode (GCE). Composite materials can be defined as a combination of two or more materials which are physically distinct, dispersed in a controlled way to achieve optimum properties and in which the properties are superior to those of the individual components [70]. Furthermore, the International Union of Pure and Applied Chemistry (IUPAC) defines nanocomposites as composite materials in which at least one of the phases has at least one dimension of the order of nanometers [71]. Under DPV optimized conditions, the as-prepared GCE/rGO-AuNPs electrode was successfully applied to quantify the referred antibiotic in synthetic effluent samples, even in the presence of other potential organic interferences (estriol, carbaryl, and trimethoprim). Only the carbaryl pesticide influenced the recovery tests (93.91–109%) because it oxidizes close to the potential of the target molecule. Chen et al. [72] developed a promising alternative for point-of-care monitoring of sulfamethoxazole, using rGO modified screen-printed electrodes (SPE) as analytical tools. The main innovation of this proposal is the use of ascorbic acid as a GO reducing agent and agglomeration inhibitor since this reagent weakens the stacking and van der Waals interactions between graphene flakes. Associating SPE/rGO and DPV, the authors obtained stable and accurate measurements, enabling a limit of detection (LOD) of 0.04 µmol L^‒1^ sulfamethoxazole and high yields for tests performed in lake water and tap water.

**Table 1:** Graphene-based electrochemical (bio)sensors for emerging pharmaceutical pollutants in aquatic species and environmental samples.

Analyte	(Bio)Sensor	Detection Technique	Linear Range (µmol L^−1^)	Sensitivity	LOD (µmol L^−1^)	Real Sample	Ref.
**Antibiotics**							
ciprofloxacin	GCE/Pd-PAAS-GO	DPV	0.18–10.8	0.867	0.0045	shrimp sea cucumber	[68]
sulfamethazine	GCE/rGO-AuNPs	DPV	0.5–6.5	0.32	0.1	wastewater	[69]
sulfamethoxazole	SPE/rGO	DPV	0.5–50	0.235	0.04	lake water	[72]
tetracycline, chlortetracycline doxycycline oxytetracycline	SPE/rGO	AdS-DPV	20–80	0.021	12	river water	[73]
tetracycline	GCE/graphene/L-cysteine	DPV	8.0–140	0.027	0.12	tap water river water lake water	[74]
metronidazole	GCE/PDDA-graphene/L-cystine	CV	0.01–1 70–800	0.492 0.084	0.0023	lake water	[75]
metronidazole	GCE/AgNPs-graphene	LSV	0.05–10 10–4500	0.169 0.040	0.028	lake water	[76]
metronidazole	GCE/graphene-polythionine	DPV	0.05–70 70–500	0.233 0.061	0.001	tap water river water lake water	[77]
metronidazole	GCE/graphene-PDDA/DNA	LSV	0.05–100 400–9500	0.471 0.024	0.024	lake water	[78]
metronidazole	GCE/sulfonated graphene/AgNPs	DPSV	0.10–20.0	1.80	0.05	shrimp	[79]
chloramphenicol			0.02–20.0	1.94	0.01		
**Anti-Inflammatories**							
acetylsalicylic acid	Pt/rGO-AuNPs	DLSV	0.88–2.80	-	0.26	wastewater	[80]
piroxicam	GCE/ZnO-GO-glutathione	DPV	0.1–100 100–500	0.206 0.030	0.0018	tap water	[81]
diclofenac	GCE/graphene-PDDA	DPV	20–100 20–200	0.071	0.609	lake water	[82]
acetaminophen				0.636	0.221		
diclofenac	FTO/graphene-CdS/AuNPs/aptamer	PhotoAMP	0.001–0.15	4.32	0.00078	lake water	[83]
**Hormones**							
17β-estradiol	GCE/rGO-MIP-Fe_3_O_4_	DPV	0.05–10	0.871	0.000819	water	[84]
17β-estradiol	GCE/rGO-CuTthP	DPV	0.1–1.0	0.510	0.0053	river water	[85]
17β-estradiol	Au/MCH/graphene-aptamer	DPV	7.0 × 10^−8^–1.0 × 10^−5^	-	5.0 × 10^−8^	tap water	[86]
17β-estradiol	GCE/graphene-PANI/ PAMAM-Au/ antigen/HRP-Ab-GO	DPV	0.0272–0.272	-	0.0272	tap water	[87]
17β-estradiol	GCE/exfoliated graphene	DPV	0.010–1.5	4.43	0.0049	lake water	[88]
diethylstilbestrol			0.025–3.0	1.65	0.01087		
diethylstilbestrol	SPE/GQD	LSV	0.05–7.5	0.236	0.0088	tap water	[89]
17α-ethinylestradiol	SPE/rGO/paper/SNPs/Ab	SWV	1.69 × 10^−6^–6.10 × 10^−4^	-	3.37 × 10^−7^	river water tap water	[90]

Ab—antibody; AdS-DPV—adsorptive stripping differential pulse voltammetry; AgNPs—silver nanoparticles; AMP—amperometry; AuNPs—gold nanoparticles; CV—cyclic voltammetry; CuTthP—Cu(II)-meso-tetra(thien-2-yl) porphyrin; DLSV—derivative linear scan voltabsorptometry; DPV—differential pulse voltammetry; DPSV—differential pulse stripping voltammetry; FTO—fluorine doped tin oxide; GCE—glassy carbon electrode; GO—graphene oxide; GQD—graphene quantum dots; HRP—horseradish peroxidase; LSV—linear sweep voltammetry; MCH—6-mercapto-1-hexanol; MIP—molecularly imprinted polymer; PAAS—polyacrylate; PAMAM—poly(amino-amine) dendrimers; PANI—polyaniline; PDDA—poly(diallydimethylammonium chloride); rGO—reduced graphene oxide; SNPs—silica nanoparticles; SPE—screen-printed electrode.

Thinking about disposable and portable devices for in situ analysis, Lorenzetti et al. [73] also used SPE/rGO to determine the total content of tetracyclines (tetracycline, chlortetracycline, doxycycline, and oxytetracycline) in river water by adsorptive stripping differential pulse voltammetry (AdS-DPV). rGO enhanced the electroanalytical signal by increasing the active area of the device, but the method has a short linear range (20–80 µmol L^‒1^) and relatively low inter-electrode precision (RSD = 18%), indicating that this proposal still needs to be refined to increase its applicability. Better results were found by Sun et al. [74] when employing a GCE/graphene/L-cysteine platform to quantify tetracycline. Both modifiers promoted electronic conduction, reducing the charge-transfer resistance assessed by electrochemical impedance spectroscopy (EIS). This synergistic effect contributed for the amplification and catalysis of the electroanalytical signal, justifying the more attractive results in comparison to the system mentioned earlier. By monitoring tetracycline oxidation on GCE/graphene/L-cysteine by DPV, a linear range from 8.0 to 140 µmol L^‒1^, LOD = 0.12 µmol L^‒1^, and RSD = 4.03% for inter-sensor precision tests were obtained. The proposed electroanalytical method was successfully applied in the quantification of the analyte in tap water, river water, and lake water, attesting its efficiency.

**Figure 1:**
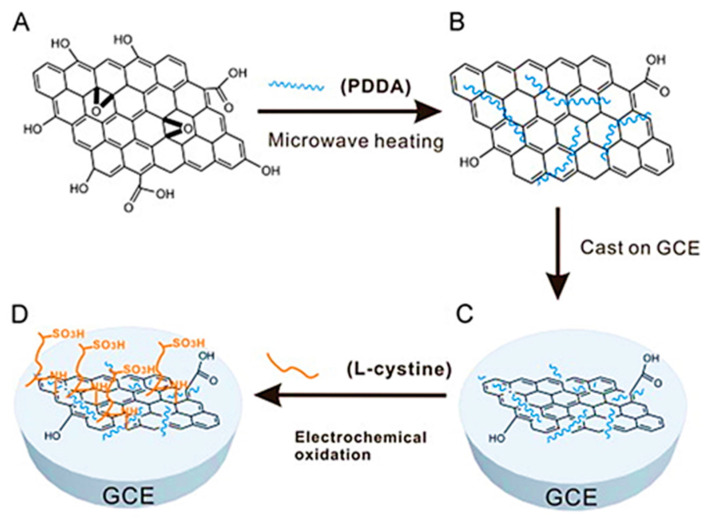
Layout of graphene functionalization and development of L-cystine/PDDA-graphene/GCE electrochemical sensor-(**A**) exfoliated graphene oxide, (**B**) poly(diallydimethylammonium chloride)-functionalized graphene obtained by a microwave-heating procedure, (**C**) functionalized graphene modified GCE, and (**D**) immobilization of L-cystine on functionalized graphene modified GCE by electrochemical grafting (reproduced from [75], with permission from Elsevier, 2012).

Seeking an alternative for monitoring the antibiotic metronidazole in biological fluids and lake water, Liu et al. [75] used a composite film derived from cysteic acid and poly(diallydimethylammonium chloride)-functionalized graphene as electrode material for ultrasensitive determination of metronidazole. The steps from graphene functionalization to sensor development are shown in Figure 1. This modifier was indispensable to record a well-defined reduction peak that was used to determine this antibiotic within different linear ranges (0.01–1 µmol L^−1^ and 70–800 µmol L^−1^), with a LOD of 2.3 nmol L^−1^. Li et al. [76] produced petal-like graphene-silver nanoparticle (AgNPs) composites with highly exposed active edge sites for the same purpose. Physicochemical characterization indicated that petal-like graphene is an ideal nucleation material for AgNPs deposition through the modified silver mirror reaction, without destroying its intrinsic structural properties. The quantification of metronidazole on GCE/graphene/AgNPs was also performed within bimodal linear ranges, however achieving a ten times higher LOD value. In turn, Yang et al. [77] managed to achieve the lowest LOD value (1 nmol L^−1^) for the same drug using an electrochemical sensing platform based on three-dimensional graphene-like carbon architecture and polythionine which was applied in several type of waters. Among other advantages, the authors highlighted the 3D porous structure, large electroactive surface area, excellent electrical conductivity, and electrocatalytic effect of the nanostructured modifier towards metronidazole reduction. The selectivity of the electroanalytical method in the presence of different cations (Mg^2+^, Na^+^, and K^+^), anions (SO_4_^2−^, NO_3_^−^, PO_4_^3−^, and CH_3_COO^−^) and organic compounds (glucose, acetaminophen, *p*-nitrophenol, *p*-aminophenol, *o*-aminophenol, imidazole, benzimidazole, and 2-methylimidazole) reiterated its viability. Despite the many positive points found in the electrochemical sensors mentioned above, the simultaneous electroanalytical determination of antibiotics remains an important barrier to overcome. In this context, a promising platform (GCE/sulfonated graphene/AgNPs) to determine chloramphenicol and metronidazole in parallel was reported by Zhai et al. [79]. Application tests performed with shrimp samples showed that the proposed sensor achieved recoveries comparable to the chromatographic standard method. Clearly, the first option (sensor) is more attractive in terms of speed, cost, operationality, and waste generation.

Graphene-based electrochemical (bio)sensors are also especially useful for electroanalysis of non-steroidal anti-inflammatory drugs. Such compounds are often sold over-the-counter, facilitating the practice of self-medication by consumers and consequently increasing their release into the environment. Regarding this type of compounds, Prado et al. [80] reported a spectroelectrochemical study focused on monitoring acetylsalicylic acid, using a platinum electrode modified with rGO-AuNPs as working sensor (Pt/rGO-AuNPs). The hybrid modifier improved the sensitivity of the device, allowing determination of low analyte concentrations even in the presence of potential electroactive interferences, such as ascorbic and uric acid. The method was successfully applied to analyze the drug in wastewater through a simple, fast and direct protocol. Dhanalakshmi et al. [81] engineered a glutathione grafted GO-ZnO nanocomposite that enhanced piroxicam sensing. The modifier’s nano-size arrangement provided a remarkable amplification and electrocatalysis of the analyte oxidation signal since this material facilitates the charge-transfer processes by increasing the active area of the device. Using GCE/ZnO-GO-glutathione under optimized voltammetric conditions, it was possible to quantify piroxicam at nanomolar concentrations in water samples with good recoveries. These results were cross-checked by high-performance liquid chromatography. Okoth et al. [82] developed a sensor for simultaneous determination of diclofenac and acetaminophen using poly(diallyldimethylammonium chloride)-functionalized GCE/graphene. During drug analysis, the authors realized that graphene enhanced the electrooxidation peak currents, while the positively charged polyelectrolyte provided well-separated peak potentials. The proposed electrochemical sensor achieved high sensitivities for both drugs, being suitable for their analysis in lake water.

**Figure 2:**
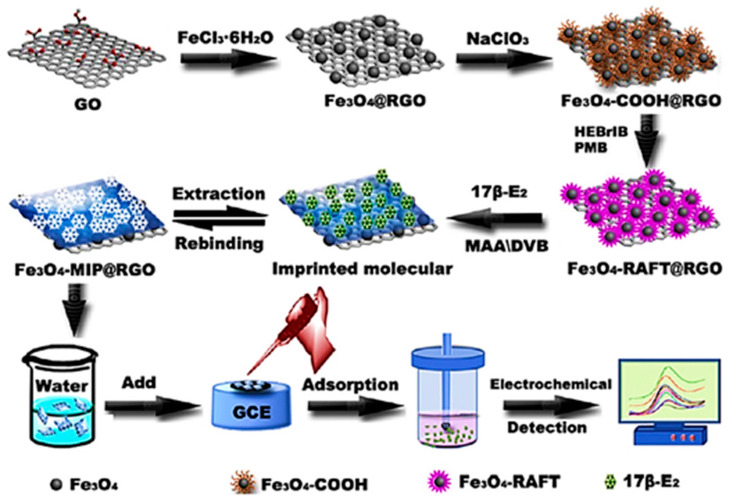
General scheme of Fe_3_O_4_-MIP-rGO/GCE sensor development and electroanalytical signal related to the oxidation of 17β-estradiol (reproduced from [84], with permission from Elsevier, 2015).

Also, synthetic and natural hormones are widely spread in the environment and therefore object of considerable study from graphene-based (bio)sensors. Looking for alternative technologies to monitor 17β-estradiol in aqueous matrices, Li et al. [84] identified an impressive sensitivity (LOD = 0.819 nmol L^‒1^) when working with Fe_3_O_4_ nanobeads-rGO/GCE as a novel molecularly imprinted (MIP) sensor. A general scheme from the nanocomposite production to the recording of the sensor signal is shown in Figure 2. Naturally, the double-layer capacitance changes as deposition of modifiers is performed, but the system became more resistive after immobilizing the non-conducting MIP membrane on the transducer surface. In contrast, the abundant imprinted cavities allowed the selective 17β-estradiol diffusion through this membrane and preserved an expressive electrochemical response in the presence of Fe_3_O_4_ nanobeads-rGO nanocomposite. Moraes et al. [85] also proposed a sensitive sensor for 17β-estradiol based on Cu(II)-*meso*-tetra(thien-2-yl)porphyrin supported over the rGO surface. Though the LOD value was about 6-times higher than the previous study, MIP-based sensors have significant hysteresis since the template is irreversibly bond to the imprinted sites and cannot be desorbed without additional treatment.

Still commenting on the negative impacts of non-steroidal hormones, a worldwide concern on development of sensors for diethylstilbestrol in the most diverse matrices exists. This compound has a stilbene-like estrogen structure and it was identified as the first scientifically proven endocrine-disrupting drug, which reinforces the need for its monitoring. Gevaerd et al. [89] developed a simple methodology for sensing of diethylstilbestrol using disposable graphene quantum dots (GQD/SPE) platforms. This novel material has exceptional electrochemical and optical properties which are advantageous for electrochemical biosensors as highlighted in a recent review [91]. The proposed sensor achieved suitable sensitivity (LOD = 8.8 nmol L^−1^) and precision (RSD < 5.0% for repeatability tests) to quantify the analyte in tap water spiked samples, enabling good recoveries. Hu et al. [88] used graphene prepared via one-step exfoliation in N-methyl-2-pyrrolidone as active material to determine diethylstilbestrol and 17β-estradiol simultaneously with similar LOD values compared with the previous sensor. On the surface of liquid-phase exfoliated graphene/GCE, an independent and greatly-increased oxidation wave was observed for each compound, showing the importance of the nanostructured modifier in the sensor configuration. The obtained analytical performance to detect diethylstilbestrol and 17β-estradiol in lake water samples were satisfactory, also presenting excellent recoveries (99.0–104.4%). However, the authors pointed out that the graphene-modified GCE was unqualified for successive measurements due to surface fouling and continuous suppression of oxidation peaks, i.e., a new sensor was required for each measurement.

Biosensors based on immunological and deoxyribonucleic acid (DNA) elements perform target biorecognition with high affinity and specificity, usually achieving low LODs in the order of pico- to femto- mol L^−1^. For example, a graphene-based aptasensor developed for 17β-estradiol reached a notable LOD of 1 × 10^−8^ µmol L^−1^ being the lowest value of the graphene (bio)sensors presented in Table 1. In this proposal, a gold electrode with a self-assembled monolayer of 6-mercapto-1-hexanol was further modified with a dispersion complex of graphene and 17β-estradiol aptamer. Here, graphene serves as attachment platform for the aptamer and as well as signal enhancer. Tap water samples were taken and spiked in order to validate the aptasensor [86]. In turn, the immunosensor for the same hormone developed by Li et al. [87] involved a more cumbersome endeavor by using a graphene-polyaniline (PANI) complex, a ethylenediamine-core poly (amidoamine)-gold nanoparticles (PAMAM-AuNPs) composite and horseradish peroxidase-graphene oxide-antibody (HRP-GO-Ab) conjugates, but obtained instead a fairly LOD value of 0.027 µmol L^−1^. Proper validation was also performed in spiked tap water samples [87]. The presence of the synthetic hormone 17α-ethinylestradiol in river and tap water was also studied by a graphene-based immunosensor [90]. This method is based on a pre-concentration step occurring in a paper substrate containing silica nanoparticles (SNPs) and the hormone antibody. The paper substrate is then placed on top of a SPE modified with rGO where 17α-ethinylestradiol is detected after desorption with diluted H_2_SO_4_. The immunocapture and pre-concentration procedures revealed an outstanding analytical performance by achieving an outstanding LOD of 3.37 × 10^−7^ µmol L^−1^, which enabled positive determinations of 17α-ethinylestradiol in all the real samples analyzed.

In a brief overview of Table 1 it can be easily observed that GCE transducers were selected in most studies as a substrate for subsequent modifications as they are widely used in electrochemistry due to their robustness, well characterized surface properties, and easy modification. Alternatives to their use were based on Pt [80], Au [86], FTO [83], and on the miniaturized and versatile 3-in-1 screen-printed electrodes [72,73,89,90]. Regarding the detection technique, DPV was frequently employed due to its superior sensitivity, followed by LSV. Also, as a note, in most studies graphene was obtained through graphite processing accordingly to a modified Hummer’s method despite being commercially available as dispersion solution or powder, in oxidized or reduced form. This manufactured method consists in the synthesis of graphene oxide through chemical oxidation of natural graphite by using NaNO_3_ and KMnO_4_ in concentrated H_2_SO_4_ or by conjugating with different oxidizers in order to prevent toxic by-products and increase yield [92].

It is worth mentioning that although graphene-based (bio)sensors demonstrate a set of advantages for screening emerging pharmaceutical pollutants, there are other carbon nanostructures that also present satisfactory results for the same purpose, either alone or in association with other materials, as will be discussed in the next sections.

### 2.2. Carbon Nanotubes

Carbon nanotubes (CNT) in the form of individual (Single-walled carbon nanotubes, SWCNT) or multiple concentric tubes (multi-walled carbon nanotubes, MWCNT) are well-established in the (bio)sensor field as valuable nanomaterials given their relevant electronic and mechanical properties [93,94,95]. Although there is not a strict consensus, the remarkable electrocatalytic behavior of CNT can be associated with edge-plane like sites present in the open ends and in defect areas along the surface or attributed to reactive groups or metallic impurities present in their structure [96,97,98]. Additionally, CNT surfaces are easily modified with different functional groups which facilitates the attachment of biological entities to develop biosensors. Therefore, all these characteristics have been extensively used in the development of electrochemical (bio)sensors [46,99,100,101,102,103].

About 26 works have employed CNT, solely or in combination with other non-carbon nanomaterials, in the (bio)sensor assemblies that were applied for the detection of a total of 25 different pharmaceuticals from 6 classes. The type of nanostructured modification, its characteristics, and analytical performance are presented in Table 2. In general, the detection of pharmaceuticals was performed through diverse electrochemical techniques with DPV being the most frequently used technique.

According to Table 2, the synthetic hormone 17α-ethinylestradiol, was the most studied drug by CNT-based (bio)sensors. Regarding this hormone, the (bio)sensor with the highest sensitivity (LOD of 3.4 × 10^−8^ µmol L^−1^) also coincided with the simplest one developed: a GCE modified with Nafion^®^-dispersed MWCNT [104]. This method consisted in an extraction step performed previously to detection, based on immunoaffinity principles where magnetic nanoparticles with attached antibodies were incubated in the sample containing the hormone and then recovered by a magnet and released in the electrochemical cell. During applicability tests, a concentration of 2 × 10^−5^ µmol L^−1^ was found in unspiked river water [104]. Also, a low value of LOD (0.0033 µmol L^−1^) for this hormone was achieved by the sensor presented recently by Nodehi et al. [105], which combined the synergetic effect of MWCNT, metal oxide nanoparticles (Fe_3_O_4_), and AuNPs. This sensor was validated in spiked wastewater and natural water samples, but no traces of the hormone were found in unspiked samples. In the contribution presented by Liu et al. [106], a GCE/MWCNT-Nafion^®^ was further modified by electropolymerizing a transition metalloporphyrin compound (Ni(II)tetrakis(4-sulfonatophenyl) porphyrin–NiTPPS) to increase the electrocatalytic capacity of the sensor. Porphyrins and metalloporphyrins have been applied in photochemical and electrochemical applications due to their catalytic properties [107]. The detection of the hormone was conducted by amperometry at +0.7 V in a flow injection system, where a linear range from 0.2 to 60 µmol L^−1^ and a LOD of 0.12 µmol L^−1^ was obtained. Spiked samples of lake, underground well, and tap water were used to validate the sensor [106]. In a similar chemical modification, a cobalt based phthalocyanine (CoPc) was used for signal enhancement. Previously to this deposition, GCE was first modified with three different carbon materials (graphite, MWCNT, and rGO) and the performances were compared by cyclic voltammetry (CV). Although rGO and MWCNT obtained a similar peak intensity, the later was chosen to modify the GCE since the peak was better defined due to lower capacitive current. The GCE/MWCNT/CoPc was applied for the detection of 17α-ethynilestradiol in river water, first unspiked (<LOD) and then spiked with known amounts in order to validate the method [108]. Taking in consideration all the (bio)sensors developed for 17α-ethinylestradiol, this last sensor displayed the least sensitivity (higher value of LOD) probably due to a lower level of nanostructuration of the surface when compared to the other works.

As can be seen in Table 2, GCE was widely used as electrode of choice, with a few exceptions. Systematically, their surface has been modified first with CNT, alone or as composites, to substantially increase the surface area, maintaining at the same time the chemical stability of the (bio)sensor and opening ways for subsequent modifications. One of the exceptions regards the analysis of acetaminophen through a gold-based electrode [109]. More specifically, a commercial digital versatile disc made of gold was cut and a nanocomposite mixture of MWCNT and copper nanoparticles (CuNPs) was deposited on its surface. At optimized pH 9, the sensor operated linearly from 0.5 to 80 µmol L^−1^ and achieved a LOD of 0.01 µmol L^−1^ in spiked wastewater and river water samples. It is important to emphasize the pertinence of this sensor for environmental analysis as its disposable and low-cost characteristics make it suitable for in-situ analysis. Two other acetaminophen sensors were developed, however, with lower performance [110,111]. In the first case, a carbon paste electrode of MWCNT and mineral oil was used for the simultaneous detection of acetaminophen and three other phenolic compounds (hydroquinone, catechol, and 4-nitrophenol) by DPV in the presence of a cationic surfactant in order to promote electron transfer by lowering the detection overpotential and increasing the peak currents. The sensor was then successfully tested in spiked tap water and domestic wastewater samples achieving good recoveries [110]. The other sensor was based on a more effortful strategy by modifying a GCE with tetraaminophenyl porphyrin (TAPP) functionalized MWCNT and AuNPs for the simultaneous detection of acetaminophen and *p*-aminophenol. Although obtaining a wide linear range, the sensitivity achieved was inferior compared with the two previous described acetaminophen sensors. River water samples were directly analyzed for the drug content by DPV with no traces being found however no traces of the drug were found [111]. Noteworthy and unusually, a boron-doped diamond (BDD) electrode was also modified simply by depositing Nafion^®^-dispersed MWCNT and employed as transducer. This sensor was applied for the detection of the antibiotic drug, ciprofloxacin, obtaining a high sensitivity with a corresponded LOD of 0.005 µmol L^−1^ through DPV. Wastewater samples were first evaluated for ciprofloxacin content (<LOD) and further used for validation of the sensor [112]. In turn, the sensor developed by Garrido et al. [113] for ciprofloxacin consisted in the modification of a GCE, first with electropolymerized aniline (PANI) and then with a mixture of MWCNT and β-cyclodextrin. This combines faster electron transfer of MWCNT with the chemical recognition of guest molecules offered by cyclodextrins [114]. The sensor attained a good LOD value of 0.05 µmol L^−1^, though being 10 times higher than the previously described sensor [112]. This can be probably explained, in part, using CV for screening of the drug, which usually shows lower sensitivity when compared with square-wave voltammetry (SWV) and DPV. Also in this study, wastewater was used as real sample to further validate the sensor [113].

**Table 2:** Carbon nanotubes-based electrochemical (bio)sensors for emerging pharmaceutical pollutants in aquatic species and environmental samples.

Analyte	(Bio)Sensor	Detection Technique	Linear Range (µmol L^−1^)	Sensitivity (µA µmol^−1^ L)	LOD (µmol L^−1^)	Real Sample	Ref.
**Anti-Inflammatories**							
acetaminophen	Au/MWCNT-CuNPs	DPV	0.5–80	2.79	0.01	Wastewater river water	[109]
acetaminophen	CPE (MWCNT)	DPV	15–180	0.414	0.29	tap water wastewater	[110]
acetaminophen	GCE/MWCNT-CONH-TAPP/AuNPs	DPV	4.5–500	0.038	0.44	river water	[111]
diclofenac	GCE/Cu(OH)_2_-IL-MWCNT	DPV	0.18–119	0.015	0.04	sea water fish serum	[115]
diclofenac	GCE/MWCNT-AuNPs/antigen/ GO-g-C_3_N_4_-Ab	ECL	0.000017–3.4	–	5.7 × 10^−6^	tap water, lake water, wastewater	[116]
diclofenac	Au/AuNPs/MWCNT-GO	DPV	0.4–80 100–1000	0.037 0.019	0.09	tap water	[117]
ibuprofen	GCE/Chit-IL-MWCNT/TPA/aptamer/MB	DPV	0.00007–6	-	0.00002	wastewater	[118]
salicylic acid	CNT-epoxy composite	SWV	200–1200	0.073	4	river water tap water	[119]
**Antibiotics**							
ciprofloxacin	BDD/MWCNT-nafion	DPV	0.005–0.05 0.05–10	41 2.08	0.005	wastewater	[112]
ciprofloxacin	GCE/PANI/βcyclodextrin-MWCNT	CV	10–80	0.257	0.05	wastewater	[113]
tetracycline	GCE/MWCNT-nafion	AMP	2.5–100	-	0.12	fish farm water well water	[120]
oxytetracycline			2.5–100	-	0.09		
chlortetracycline			1–100	-	0.31		
doxycycline			1–100	-	0.44		
oxytetracycline	GCE/MWCNT-Chit/IL/AuNPs	AMP	0.2–9	-	0.02	fish meat	[121]
metronidazole	GCE/MWCNT/MIP	CV	0.001–1.2	-	0.00029	fish meat	[122]
sulfanilamide	GCE/MWCNT-nafion	AMP	0.6–58.1	1	0.058	river water tap water	[123]
sulfadiazine			0.4–20	4.38	0.16		
sulfaguanidine			0.5–46.7	3.55	0.14		
sulfathiazole			0.4–19.6	12.27	0.118		
sulfamerazine			0.4–11.4	2.65	0.076		
sulfisoxazole			1.9–37.5	2.99	0.037		
sulfamethoxazole	CPE (MWCNT-SbNPs)	DPV	0.1–0.7	0.57	0.024	natural water (dam)	[124]
trimethoprim			0.1–0.7	0.37	0.031		
sulfamethazine	GCE/SWCNT-Chit/CTAB-AuNPs/antigen/Ab1/Ab2-AgNPs-DFNS	AMP	0.0016–0.15	-	0.00024	river water lake water pond water	[125]
**Hormones**							
17α-ethinylestradiol	GCE/MWCNT-nafion/PolyNiTPPS	AMP	0.2–60	0.12	0.12	well water tap water lake water	[106]
17α-ethinylestradiol	GCE/MWCNT-Nafion	SWV	6.8 × 10^−8^–2.4 × 10^−4^	-	3.4 × 10^−8^	river water tap water	[104]
17α-ethinylestradiol	GCE/SWCNT-Cdot/laccase	DPV	0.05–7	0.75	0.004	tap water	[126]
17α-ethinylestradiol	GCE/MWCNT/CoPc	SWV	2.5–90	0.179	2.2	river water	[108]
17α-ethinylestradiol	GCE/MWCNT/Fe_3_O_4_-Tannic_acid-AuNPs	DPV	0.01–120	0.676	0.0033	Wastewater natural water	[105]
diethylstilbestrol	GCE/AuNPs/MWCNT-CoPc	SWV	0.79–5.7	1.05	0.2	natural water (dam)	[127]
17β-estradiol	GCE/MOF(Al)-CNT/PB/MIP(PPy)	DPV	1 × 10^−8^–1 × 10^−3^	21000	6.2 × 10^−9^	pond water	[128]
**Anticonvulsants**							
carbamazepine	GCE/MWCNT	LSV	0.13–1.6	2.59	0.04	wastewater	[129]
**Antiacids**							
omeprazole	GCE/PDDA/Fe_3_O_4_-MWCNT	LSV	0.05–9	3.28	0.015	wastewater	[130]
**β-Blockers**							
propranolol	GCE/MWCNT-IL	DPV	–	–	–	lake water	[131]

Ab—antibody; AgNPs—silver nanoparticles; AMP—amperometry; Au—gold; AuNPS—gold nanoparticles; BDD—boron-doped diamond electrode; Cdot—carbon dot; Chit—chitosan; CNT—carbon nanotubes; CONH-TAPP—tetraaminophenyl porphyrin; CoPc—cobalt phthalocyanine; CPE—carbon paste electrode; CTAB—cetyltrimethylammonium bromide; CuNPs—copper nanoparticles; CV—cyclic voltammetry; DFNS—dendritic fibrous nanosilica; DPV—differential pulse voltammetry; ECL—electrochemiluminescence; GCE—glassy carbon electrode; g-C3N4—graphite-like carbon nitride; GO—graphene oxide; IL—ionic liquid; LSV—linear sweep voltammetry; MB—methylene blue; MIP—molecularly imprinted polymer; MOF—metal organic framework; MWCNT—multi-walled carbon nanotubes; PANI—polyaniline; PB—Prussian blue; PDDA—poly(2,6-pyridinedicarboxylic acid); PolyNiTPPS—Ni(II)tetrakis(4-sulfonatophenyl) porphyrin; PPy—polypyrrole; SbNPs—antimony nanoparticles; SWCNT—single-walled carbon nanotubes; SWV—square-wave voltammetry.

Interestingly, from the 26 studies presented in Table 2, only two resorted to SWCNT for (bio)sensor enhancement [125,126]. Since both MWCNT and SWCNT have similar electrocatalytic properties, the later nanostructure is lesser used probably due to lower synthesis yield and higher production costs [132]. In this respect, Canevari et al. [126] used an enzymatic biosensor for 17α-ethynilestradiol determination. To promote direct electron transfer between the transducer surface (GCE) and the copper atoms of laccase active site, a hybrid nanocomposite of SWCNT and carbon dots (Cdot) was used taking advantage of the π-interactions between these two materials. The GCE/SWCNT-Cdot/laccase exhibited a DPV peak at +0.1 V achieving an acceptable LOD of 0.004 µmol L^−1^. The stability of the film containing the biological entity can be a critical parameter in biosensors. Particularly in this case, the reproducibility achieved was about 3% for five different electrodes and moreover, it was stable for 1 month in storage without signal loss. The biosensor was ultimately validated in spiked tap water with recoveries of about 100%. Whereas, the SWCNT-based biosensor developed by Yang et al. [125] relied in a competitive immunoassay intended for environmental screening of the antibiotic drug sulfamethazine. In order to amplify the electrochemical signal, a first layer of SWCNT (in the form of nanohorns) dispersed in chitosan (Chit) was deposited on a GCE surface followed by a layer of cetyltrimethylammonium bromide-capped gold nanoparticles (CTAB-AuNPs). Antigen, the drug, and the antibody were then sequentially deposited, and the surface was finally modified with silver nanoparticle functionalized with dendritic fibrous nanosilica (AgNPs-DFNS). With the increase of the drug concentration, less AgNPs were available in the biosensor surface leading therefore to a proportional decrease in the H_2_O_2_ catalytic response. The immunosensor achieved a low value of LOD corresponding to 2.4 × 10^−4^ µmol L^−1^ and it was finally validated in spiked river, lake, and pond waters achieving satisfactory recoveries (79–118%), though the intra-assay variation was up to 10% for this biosensor [125]. Other CNT-based sensors were developed for different sulfonamide drugs [123,124]. For instance, six drugs belonging to this group of antibiotics—namely sulfanilamide, sulfadiazine, sulfaguanidine, sulfathiazole, sulfamerazine, and sulfisoxazole—were studied individually by a GCE/MWCNT-Nafion^®^ sensor [123]. Amperometric detection of all drugs in river and tap water samples was conducted in a flow system and revealed an overall good sensitivity, with LOD ranging from 0.037 µmol L^−1^ (sulfisoxazole) to 0.160 µmol L^−1^ (sulfadiazine) [123]. In the other proposal, the drugs sulfamethoxazole and trimethoprim (which are often associated in the same pharmaceutical prescription) were simultaneously detected in natural water collected from a dam. A carbon paste sensor was made, taking advantage of the synergistic effect of both MWCNT and antimony nanoparticles (SbNPs), mixed with paraffin. Using DPV, the obtained LODs for both drugs were in the same order of magnitude compared with the previous described sensor [124]. Although not as competitive as biosensors in terms of analytical performance, these last presented sensors from Bueno et al. [123] and Cesarino et al. [124] offers simplicity and rapidness in sensor assembly with fairly good sensitivity, representing favorable features.

Regarding the type of the environmental samples analyzed, the majority of the (bio)sensors were applied in water samples collected in rivers, lakes, wastewaters, and ponds. Nevertheless, two studies have opted to analyze antibiotics in fish tissues since this class of pharmaceuticals is widely used in aquaculture as veterinary drugs and therefore is susceptible to be found in these type of samples. Naggles et al. [121] were able to detect the oxytetracycline antibiotic in trout fish. The sensor consisted in different layers of conductive materials, namely, MWCNT dispersed in Chit, ionic liquid (IL), and electrodeposited AuNPs (GCE/MWCNT-Chit/IL/AuNPs). Amperometric detection was revealed to be more efficient compared with the adsorptive stripping SWV technique by achieving a lower LOD (0.02 µmol L^−1^) and therefore it was used in the real fish trout samples [121]. The detection of positive samples proved the applicability of this sensor and the possibility of contamination occurrence in fish farms. Likewise, metronidazole was investigated in carp fish with a selective sensor that was obtained by coupling the increased surface area and electrical properties offered by MWCNT, and the recognition ability of MIP technology based on electropolymerized dopamine [122].

**Figure 3:**
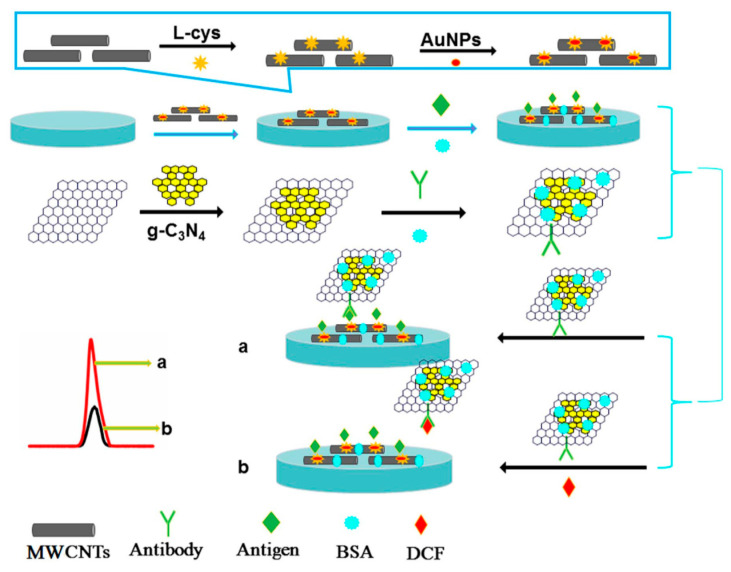
Procedure assembly scheme of a electrochemiluminescent immunosensor for diclofenac detection consisting on a GCE/MWCNTs-AuNPs/coating antigen/BSA/GO-g-C_3_N_4_ labeled DCF antibody (reproduced from [116], with permission from Elsevier, 2017).

The conjugation of MWCNT with other carbon nanomaterials, namely graphene, was considered in two different studies for detection of the same drug, the widely used anti-inflammatory diclofenac [116,117]. In one of the proposals [116], a electrochemiluminescent (ECL) immunosensor was fabricated by first modifying a GCE with a synthesized MWCNT-AuNPs nanocomposite. After coating with antigen, a second composite of graphene oxide and graphitized carbon nitride (GO-g-C_3_N_4_) with attached diclofenac antibodies was deposited alongside different concentrations of diclofenac. In this competitive immunoassay, diclofenac present in a solution compete with the coated antigen for the limited sites of antibodies therefore changing the generated ECL signal. Thus, in the absence of diclofenac the signal is higher (Figure 3). The authors attributed the high performance of this biosensor to the effective immobilization of a large amount of coating antigen onto the MWCNTs-AuNPs and due to the ECL intensity enhancement promoted by GO-g-C_3_N_4_. Three different waters—namely, tap, lake and wastewater—were analyzed for diclofenac content. However, only in wastewater samples was diclofenac detected above LOD with a mean concentration of 0.0033 µmol L^−1^ [116]. According to the literature, 3D network of nanocomposite based on MWCNTs-GO combined the high charge density of GO with a high surface area of MWCNT providing a highest edge density per unit area, promoted the enhancement of electron transfer process. Furthermore, metallic nanoparticles can be used combined com carbon nanocomposites to further improve the electroanalytical properties. In this context, the other carbon nanocomposite proposal consisted in a gold electrode modified with a MWCNT/GO nanocomposite film and AuNPs electrochemically deposited [117]. At optimized conditions, the diclofenac calibration curve, obtained by DPV measurements, revealed a wide linear range and reached a LOD of 0.09 μmol L^−1^ [117], which is a comparably higher value than the previous carbon composite immunosensor [116]. Tap water was analyzed as real sample with no traces of diclofenac being found [117].

It seems evident that the most sensitive (bio)sensors are based in highly specific recognition elements such as MIP [122,128] or biological entities involving aptamers [118] and antibodies [104,116,125]. In this way, the lowest LOD (6.2 × 10^−9^ µmol L^−1^) corresponded to a MIP sensor developed for 17β-estradiol detection in environmental waters. In fact, a 17β-estradiol concentration of 4 × 10^−8^ µmol L^−1^ was determined in unspiked pond water without pre-concentration or pre-treatment of the sample, revealing environmental contamination at trace level. The MIP sensor was assembled by first modifying a GCE with an aluminum-based metal–organic framework (MOF) containing in the dispersion media CNT to improve MOFs electronic properties. Further modification was performed by electrodeposition of a Prussian blue film and polypyrrole (PPy) as the imprinting polymer [128]. Specially in biosensors and MIP design, the importance of CNT structures is evident in the ability to increase load capacity, biocompatibility, and chemical stability, which seem more relevant features than electrochemical signal enhancement.

Overall, (bio)sensor construction relies on other materials in addition to CNT to enhance further the analytical performance. Metal and metal oxides nanomaterials are often conjugated with CNT as nanocomposites, being AuNPs the most relevant example. Also polymeric media such as Chit and Nafion^®^ have been used as effective dispersing agents, acting as well as entrapment matrices or barrier to interferents [133,134]. ILs are another component that have been advantageously used with CNT, widening the potential window and conferring higher conductivity [100]. Relatively to the origin of carbon nanotubes used in (bio)sensors fabrication and contrary to what was assessed for graphene, these were mainly commercially acquired from chemical companies rather than being produced by the authors themselves probably due to their relatively low cost and high quality. There are different methods for carbon nanotube synthesis, however chemical vapor deposition (CVD) is established as the most efficient enabling higher yields and scalability. Simply, it consists in the decomposition of gaseous hydrocarbons at elevated temperatures to form solid deposit into metallic catalysts. The nanotube diameter can be controlled which permits their availability in different sizes and therefore with different properties [94].

### 2.3. Carbon Black

**Figure 4:**
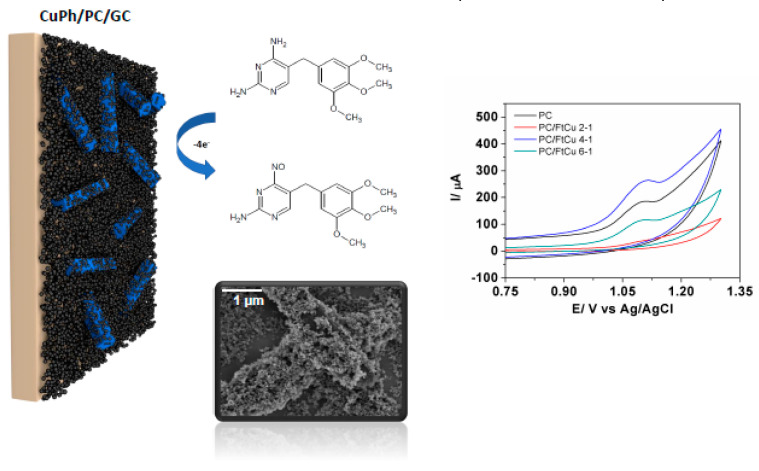
A trimethoprim antibiotic sensor composed of Printex L6 carbon black (CB) and copper (II) phthalocyanine films (reproduced from [138], with permission from Elsevier, 2018).

Carbon black (CB) has been recently explored for manufacturing transducers for emergent pollutant analysis due to some interesting advantages such as low cost, large surface area, good conductivity, and stability and the ability to form homogeneous films. This carbonaceous material can be obtained from the combustion of hydrocarbons and is composed of aggregates or agglomerates of spherical particles where each particle owns a turbostratic structure of random packing graphite layers [135], therefore having a more bulkier structure when compared with CNT or graphene. A total of eight sensors were developed for determination of nine different pharmaceuticals belonging to the classes of antibiotics, anti-inflammatories, antidiabetics, and hormones (Table 3). The class of antibiotics were also well studied by CB-based sensors. For example, Sun et al. [136] related that montmorillonite and acetylene black are effective electrocatalysts, thus improving the sensitivity of oxytetracycline determination (LOD = 0.087 µmol L^−1^) in shrimp samples. This antibiotic is used in animal husbandry and may cause the occurrence of residues in food-producing animals. The main reported highlights were the large specific area and strong adsorptive ability. Delgado et al. [137] also fabricated a sensor made with CB nanoballs and potato starch deposited onto a GCE to detect tetracycline in tap water and river water. The authors reached suitable sensitivity which was attributed to the enhanced conductivity and large area of the film. Guaraldo et al. [138] developed a sensor by modification of a GCE with Printex L6 CB nanospheres and Cu (II)-phthalocyanine for trimethoprim determination in river water (Figure 4). According to the authors, the electrocatalytic effect towards trimethoprim detection was observed due to the synergistic effect between CB-CuPc. Besides, CuPh modification resulted in strong adsorption to carbon black and trimethoprim due to π–π interactions. The analytical results obtained were two wide linear range (0.4–1.1 µmol L^−1^ and 1.5–6.0 µmol L^−1^) and a LOD of 0.67 µmol L^−1^, presenting also good reproducibility and great film stability when stored for 30 days. Deroco et al. [139] applied CB with a dihexadecylphosphate (DHP) in the manufacturing of a sensor for simultaneous detection of the antibiotic amoxicillin and the anti-inflammatory nimesulide in tap and lake waters. The DHP-CB film resulted in the enhanced promotion of the electron transfer when compared with the unmodified GCE according with the values of apparent heterogeneous electron transfer rate constant. The detection limits for amoxicillin and nimesulide were 0.12 μmol L^−1^ and 0.016 μmol L^−1^, respectively.

**Figure 5:**
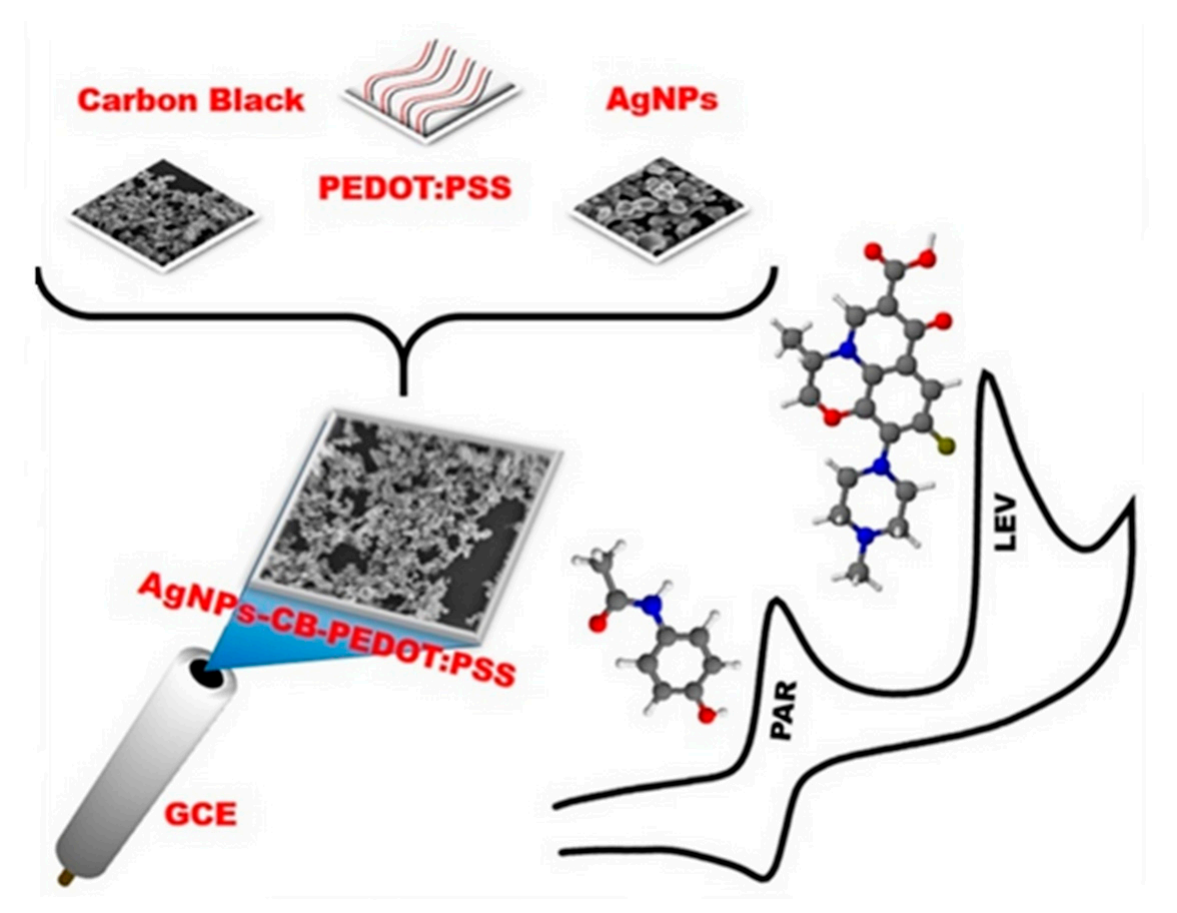
Assembly scheme of a GCE/AgNPs-CB-PEDOT:PSS sensor used for simultaneous determination of acetaminophen and levofloxacin (reproduced from [141], with permission from Elsevier, 2017).

In another study also proposed by the same research team (Deroco et al. [140]), the authors evaluated the effect of three different CB types (Vulcan XC72R, Black Pearls 4750, and CB N220) on the electrochemical behavior of levofloxacin (antibiotic) and acetaminophen (analgesic). The attained analytical signal on the SPE/CB(BP) was slightly higher due to the large number of defects and the oxygen atoms in CB(BP), which provides a larger number of active sites on the surface. SWV was used for simultaneous determination of levofloxacin and acetaminophen in river water samples, showing satisfactory results in terms of performance for both drugs. Concerning the analysis of these two compounds in river water, Wong et al. [141] modified a GCE with CB, AgNPs and poly(3,4-ethylenedioxythiophene)-poly(styrenesulfonate) (PEDOT:PSS) in order to attain a large surface area, high conductivity, and catalytic activity (Figure 5). The obtained analytical parameters with GCE/AgNPs-CB-PEDOT:PSS were much more satisfactory compared to those reported by Deroco et al. [140].

Antidiabetic drugs have been also considered as emerging contaminants, being mainly found in wastewaters. Machini et al. [142] utilized CB-dihexadecylphosphate to modify a GCE for metformin determination (LOD = 0.63 μmol L^−1^) in wastewater samples. The authors used the metformin-Cu(II) complex due to the catalytic action of Cu(II) ions towards the oxidation of metformin and the increase of current signal and the improvement of electron transfer kinetics.

Non-steroidal estrogens have been applied in livestock production, as a result diethylstilbestrol has been detected in natural water samples. A simple electrochemical device based on CB paste electrode was used for diethylstilbestrol determination in fishery water samples [143]. The method showed a LOD of 0.008 μmol L^−1^, revealing the lowest LOD obtained when compared with other sensors presented in Table 3, with moderate recoveries of 84–94% for the spiked fishery waters.

As observable in Table 3, sensors conjugating biorecognition elements with CB have not been developed so far probably owing to the more bulky graphitic structure of CB, possibly offering a lower surface area which translates in a lower loading capacity and analytical performance when compared to CNT and graphene.

**Table 3:** Carbon black-based electrochemical (bio)sensors for emerging pharmaceutical pollutants in aquatic species and environmental samples.

Analyte	Sensor	Detection Technique	Linear Range (µmol L^−1^)	Sensitivity (µA µmol^−1^ L)	LOD (µmol L^−1^)	Real Sample	Ref.
**Antibiotics**							
oxytetracycline	CPE (montmorillonite-acetylene black)	DPV	0.5–50	0.0281	0.087	fish shrimp	[136]
tetracycline	GCE/CB-PS	DPV	5–120	0.033	1.15	tap water river water	[137]
trimethoprim	GCE/CB-CuPh	SWV	0.4–1.1 1.5–6.0	5.82 0.79	0.67	river water	[138]
**Antibiotics and Anti-Inflammatories Simultaneously**							
amoxicillin nimesulide	GCE/CB-DHP	SWV	1.0–18.8 0.3–5.0	0.030 0.56	0.12 0.016	lake water tap water	[139]
levofloxacin	SPE/CB(BP)	SWV	0.90–7 0.0	0.66	0.42	river water	[140]
acetaminophen			4.0–80.0	0.31	2.6		
levofloxacin	GCE/AgNPs-CB-PEDOT:PSS	SWV	0.67–12	1.9	0.012	river water	[141]
acetaminophen			0.62–7.1	1.7	0.014		
**Antidiabetics**							
metformin	GCE/CB–DHP	DPV	2.0–10.0	640	0.63	wastewater	[142]
**Hormones**							
diethylstilbestrol	CPE(CB)	SWV	0.016–0.465	5.4	0.008	fishery water	[143]

AgNPs—silver nanoparticles; CPE—carbon paste electrode; CuPh—copper (II) phthalocyanine; DHP—dihexadecylphosphate; DPV—differential pulse voltammetry; GCE—glassy carbon electrode; PEDOT:PSS—poly(3,4-ethylenedioxythiophene)-poly(styrenesulfonate); PS—Potato starch; SPE—screen-printed electrode; SWV—square-wave voltammetry.

### 2.4. Other Carbon-Based Nanomaterials

Other carbon nanomaterials—such as mesoporous carbon [144,145], carbon nanopowder [146], biochar [147] and graphite oxide [148], fullerene and carbon nanofibers [149]—have also been utilized in the design of (bio)sensors for emerging contaminants in waters (Table 4), however with less expression. As well, antibiotics were the pharmaceutical class with more developed carbon nanostructured sensors. In this context, an amoxicillin sensor was proposed by Pollap et al. [144] based on the modification of a graphite electrode with a mixture of CMK-3-type ordered mesoporous carbon (OMC), Nafion^®^, TiO_2_ sol, and AuNPs. OMC is a highly porous carbon material with pores standing between 2 and 50 nm in diameter, accordingly to IUPAC [150]. The high surface of CMK-3 promoted the easy immobilization of AuNPs having both a positive impact on the electron transfer process with the sensor reaching a LOD of 0.3 μmol L^−1^ through CV technique. This sensor was finally validated in bottled mineral waters and river water, with the samples being spiked after no detection of residues of the drug in the samples. Lahcen et al. [146] performed a broader study in respect to the comparison of several carbon nanomaterials (CB, acetylene black, carbon nanopowder, graphite, MWCNT, and glassy carbon powder) in the formulation of paste electrodes (CPE) for detection of various sulfonamides antibiotic drugs (sulfamethoxazole, sulfadiazine, sulfacetamide, sulfadimethoxine, sulfathiazole, sulfamethiazole, and sulfamerazine). The best analytical results in controlled conditions for detection of sulfamethoxazole, as model analyte, were achieved by a CPE formulated with carbon nanopowder, showing the highest sensitivity (0.045 μA μmol^−1^ L) and the lowest LOD (0.12 μmol L^−1^). The sensor was then successful validated in drinking water obtained from a local market. A method employing graphite oxide (GrO) as main nanostructured component was developed for simultaneous detection of ofloxacin antibiotic and three other pharmaceutical drugs from different classes, namely levodopa (anti-Parkinson), piroxicam (anti-inflammatory) and methocarbamol (analgesic). Four distinctive peaks in the range of +0.4 V and +1.3 V (vs Ag/AgCl) were clearly obtained for the drugs by SWV using a carbon paste sensor (CPE) made by mixing GrO, β-cyclodextrin, and mineral oil. The obtained LODs ranged from 0.065 μmol L^−1^ (levodopa) to 0.398 μmol L^−1^ (methocarbamol). The method was finally validated in spiked river water with recoveries percentage very close to 100% [148].

Bojdi et al. [145] also resorted to a mesoporous carbon material for sensor construction. Specifically, a CPE based on the mixture of mercapto-mesoporous carbon with graphite powder and Nujol oil was used for analysis of the gastric acid inhibitor drug, omeprazole, in spiked and unspiked (< LOD) tap water samples. Calibrations curves obtained directly in the real sample by DPV revealed a linear range between 0.00025 and 25 μmol L^−1^ and a LOD of 4 × 10^−5^ μmol L^−1^. The principal advantage of mesoporous carbon is the high surface areas of modified-CPE compared to unmodified-CPE.

Dong et al. [147] prepared a sensor based on GCE modified electrode with biochar nanoparticles (biocharNPs) with high conductivity for detection of 17β-estradiol in ground water samples. The biocharNPs were obtained by slowly pyrolyzing sugarcane bagasse under nitrogen and posterior collection of the small sized particles through centrifugation. The selected nanomaterial provided ample surface area for highly efficient adsorption and enrichment of target 17β-estradiol and fast electron transfer. The figures of merit acquired by DPV technique consisted in a linear concentration range from 0.05 to 20 μmol L^−1^, LOD of 0.011 μmol L^−1^, and recoveries between 103% and 107%.

**Table 4:** Other carbon nanomaterials used in electrochemical sensors for emerging pharmaceutical pollutants in waters.

Analyte	(Bio)Sensor	Detection technique	Linear range (µmol L^−1^)	Sensitivity (µA µmol^−1^ L)	LOD (µmol L^−1^)	Real Sample	Ref.
**Antibiotics**							
amoxicillin	Graphite/TiO_2_-OMC-AuNPs-Nafion	CV	0.5–2.5 2.5–133	1.42 0.832	0.3	bottled water river water	[144]
sulfamethoxazole	CPE (CN)	SWV	1.0–10 10–75	0.045 0.017	0.12	drinking water	[146]
**Antibiotic,** **central nervous system agent, anti-inflammatory and analgesic simultaneously**							
ofloxacin levodopa piroxicam methocarbamol	CPE (βcyclodextrin-GrO)	SWV	1.0–20 1.0–15 1.0–20 1.0–50	0.550 0.551 0.699 0.075	0.065 0.105 0.089 0.398	river water	[148]
**Antiacid**							
omeprazole	CPE (mercapto-MC)	DPV	0.00025–25	1.0	0.00004	tap water	[145]
**Hormones**							
17β–estradiol	GCE/biocharNPs	DPV	0.05–20	0.85	0.0113	ground water	[147]
**Anti-inflammatories**							
acetaminophen ibuprofen	SPCNFE	DPV	–	–	0.2 2.9	tap water wastewater	[151]
diclofenac	CPE (fullereneC60-CNF)	SWV	–	1.08	0.0009	tap water	[149]

AuNPs—gold nanoparticles; biocharNPs—biochar nanoparticles; CN—carbon nanopowder; CNF—carbon nanofiber; CPE—carbon paste electrode; CV—cyclic voltammetry; DPV—differential pulse voltammetry; GCE—glassy carbon electrode; GrO—graphite oxide; MC—mesoporpus carbon; OMC—ordered mesoporous carbon; SPCNFE—screen-printed carbon nanofiber electrode; SWV—square-wave voltammetry.

In the proposal of Serrano et al. [151], a commercial screen-printed carbon electrode modified with carbon nanofibers (SPCNFE) was selected for analysis of both anti-inflammatory drugs, acetaminophen and ibuprofen, in water samples as this electrode analytically outperformed other screen-printed electrodes (unmodified and modified with MWCNT and graphene). Both drugs (as well as caffeine) were simultaneously detected by DPV exhibiting widely separated peaks (about +0.4 V for acetaminophen and +1 V for ibuprofen). In acetate buffer at pH 5.5, the LOD corresponded to about 0.2 μmol L^−1^ for acetaminophen and about 2.9 μmol L^−1^ for ibuprofen. Tap water samples were then used to properly validate the method whereas a wastewater sample was taken from a hospital effluent and directly analysed for drugs content but only acetaminophen was found at a concentration of 7.7 μmol L^−1^.

Motoc et al. [149] applied a CPE composed of fullerene C60 and carbon nanofibers (CNF), acting as a pseudomicroelectrode array, to study diclofenac detection by different voltammetric and amperometric analytical techniques. The most sensitive techniques for the drug revealed to be SWV and multiple pulsed amperometry (MPA). The sensor was therefore further characterized by SWV directly in tap water obtaining a linear range between 3.4 and 16.9 μmol L^−1^ and a sensitivity of 0.38 μA μmol^−1^ L.

## 3. Overall Comparison between Reported Nanostructured Carbon-Based (Bio)Sensors

The assessment and overall comparison of the electroanalytical performance between the reported (bio)sensors is usually a difficult and challenging process given that several parameters have influence and must be taken into account, such as: transducer and type of pre-treatment given, assay conditions (pH, ionic strength, *etc*.), detection technique and its parameters (e.g., scan rate). However, principally, the modification strategy and the adopted conjugation of materials have the major impact. Concerning this review, the comparison between different types of carbon nanomaterial should be carefully done for the same drug and with equivalent modification strategies in order to prevent an inappropriate evaluation. In this context, diethylstilbestrol hormone was analyzed by graphene-, MWCNT-, and CB-based sensors. Although LOD values were similar between the graphene [GCE/exfoliated graphene [88] and SPE/GQD [89]) and CB (CPE(CB) [143]] sensors, the MWCNT sensor (GCE/AuNPs/MWCNT-CoPc [127]) exhibited a 20-times higher value. Conversely, for tetracycline, the CB sensor (GCE/CB-PS [137]) outperformed compared with graphene (GCE/graphene/L-cysteine [74]) and MWCNT (GCE/MWCNT-Nafion^®^ [120]), which obtained exactly the same LOD value (0.12 μmol L^−1^) whereas for trimethoprim the CB sensor (GCE/CB-CuPh [138]) presented also higher value of LOD (about 20-times higher) compared with the MWCNT (CPE(MWCNT-SbNPs) [124]). In turn, LOD of sensors for acetaminophen based on graphene (GCE/graphene-PDDA [82]), MWCNT [CPE(MWCNT) [110] and GCE/MWCNT-CONH-TAPP/AuNPs [111]) and CNF (SPCNFE [151]) are in line with each other, except for Au/MWCNT-CuNPs [109] that achieved an approximately 10-fold lower value, while in both CB sensors (GCE/AgNPs-CB/PEDOT:PSS [141] and SPE/CB(BP) [140]) values vary widely by 2 orders of magnitude. By analyzing the data, we can conclude therefore that there is not a clear tendency for the impact of the type of carbon nanomaterial in the reached sensitivity. Still, in general, the most sensitive methods comprised biological entities for highly specific biorecognition. For instance, biosensors with immobilized antibodies (immunosensor) or DNA/RNA fragments (aptasensor) achieved considerably lower values of LOD, reaching concentrations between 10^−5^ to 10^−8^ μmol L^−1^, either using graphene [86,90], or CNTs [116,118] as signal enhancer and substrate for biomolecules attachment. It is worth noting that only one work [126] resorted to enzymatic biorecognition of pharmaceuticals, which shows a clear gap since many pharmaceutical drugs have phenolic derivatives that can act as substrates of enzymes such as laccase, tyrosinase, or hydrogen peroxidase [152]. Still, the application of biosensors (versus sensors) is very limited, which can be mostly attributed to the higher cost and cumbersome fabrication methods. As a matter of fact, the use of biological entities to accomplish high sensing performance can be dispensed as there are alternatives attaining similar results. One example is the application of molecularly imprinted polymers. Though also fastidious, graphene- [84] and MWCNT- [122,128] based sensors that employed this technology achieved good sensitivities on the level of sub-nanomolar concentrations.

In terms of instrumentation, a common denominator between the different types of carbon nanostructured (bio)sensors presented herein is the widely use of GCE as transducer and substrate for modification. Despite its robustness and reliability, this conventional electrode is more suitable for laboratorial work rather for in-situ environmental applications due to its bulkier dimensions. In this sense, screen-printed electrodes [72,73,89,90,140,151] and the film type electrodes of gold [109] and FTO [83] present high area-to-volume ratio enabling miniaturization and portability and, therefore, are more convenient for in loco analysis. In this miniaturization process, carbon nanomaterials can be extremely useful and exceptionally reliable through the production, for example, of buckypaper type electrodes. These are thin, lightweight, and flexible carbon mat fabricated by filtration of well dispersed carbon nanomaterial [153,154,155] that can be applied advantageously in portable lab-on-a-chip platforms and miniaturized devices [156,157] ideal for costless and in-situ environmental analysis.

Sustainability from the point of view of materials and methods used can be seen as an important and competitive characteristic of electrochemical (bio)sensors compared to the more conventional analytical methods (such as chromatographic and spectroscopic techniques). For instance, in the preparation of (bio)sensors, the quantities employed in the transducer modification are usually a few microliters and with concentrations in the order of 1 or 2 mg mL^−1^. Though frequently employing organic solvents such as dimethylformamide for dispersion of carbon nanostructures and other materials, the used volumes are insignificant when compared with the processing volumes of chromatographic techniques and therefore (bio)sensor technology can be seen as more eco-friendly. Specifically considering carbon nanostructures, possible toxic effects may be caused to the environment or humans equally as other types of nanomaterials, however there is substantial evidence of microbial and enzymatic biodegradability of carbon nanotubes and graphene [158] which is an advantage over metallic nanoparticles and polymers also widely applied in (bio)sensor fabrication.

## 4. Conclusions and Future Trends

In this review, the crucial role that carbon nanomaterials have been playing in the design of (bio)sensors for screening of emerging pharmaceutical pollutants, in waters and aquatic species, was addressed.

A total of 62 studies concerning the use of carbon nanomaterials-based electrochemical (bio)sensors were contemplated in this review, being specifically categorized as follows: 21 graphene-based, 26 CNT-based, 8 CB-based, and 7 other carbon nanomaterials-based (bio)sensors. In a different perspective, 34 pharmaceutical drugs belonging to 9 different classes were analyzed by these (bio)sensors in waters and aquatic species. As expected, antibiotics, anti-inflammatories, and hormones were the classes more studied due to their high consumption and almost ubiquitous presence in the environment. It is important to highlight that the vast majority of these studies (representing about 80% of the 62) were conducted very recently (since 2015), which attests clearly the rising interest from the scientific community on this subject and on the research opportunities that are emerging. Concerning the type of samples analyzed, only a few (bio)sensors were applied for pharmaceuticals detection in animal species. This seems relevant from a public health point of view since risk assessment is preferably accomplished in aquatic animals rather than environmental waters. Furthermore, there is also a concern from the fishery and aquaculture sector as well as from the general public regarding food safety and public health since certain pharmaceutical drugs, such as hormones and anti-inflammatories, cause deleterious effects (endocrine disruption) and may bioaccumulate in animal tissues. More studies are needed in this respect and electrochemical (bio)sensors can contribute to risk assessment and thus constitute a research opportunity soon.

During the last decade, significant advances based on the synergy between carbon-based nanomaterials, recognition elements, and different transduction schemes have been promoting the development of (bio)sensors that fulfil the required electroanalytical performance. The exceptional progress in the nanomaterials field, particularly in controlling the physico-chemical properties during synthesis, has led to outstanding improvements in the (bio)sensors design. Furthermore, the conjugation of various nanomaterials and compounds in (bio)sensor assembly may increase the (bio)sensing performance; however, it can also lead to an increase in complexity and preparation time as well as in fabrication cost and, therefore, assembly and modification strategies should be carefully equated.

It is also important to outline clear limitations of electrochemical (bio)sensors that need to be tackled in the future. As known, simultaneous analysis with this technology is still restricted to a few number of compounds as they are detected in a limited potential window with limited techniques regarding signal definition. Specifically for pharmaceutical analysis, discrimination of compound derivatives from the same drug classes is very challenging. When considering pharmaceuticals analysis in complex environmental matrices (e.g., wastewaters), this becomes an even more serious issue since numerous other pollutants can exist that can interfere or be detected at the same potential as target compounds. This limitation is transversal to all types of electrochemical (bio)sensors, including the ones based on carbon nanomaterials. Still, carbon nanomaterials can definitely contribute as a part of the solution by increasing peak definition and hence, discrimination of compounds. The other part of the solution is related with chemometric analysis. This powerful discriminatory technique is still not extensively associated with electrochemical methods probably due to some inherent complexity and therefore yet to be properly explored.

As previously outlined in this review, carbon nanomaterials have already contributed significantly to miniaturization of electroanalytical devices and will definitely continue to be important in this miniaturization process to serve the purpose and goal of in-situ environmental analysis. Undeniably, carbon nanomaterials have proven to be valuable in many different applications and, for sure, have laid the foundations to play a critical role in the future in (bio)sensing systems development.

## References

[1] (2019). World Population Prospects 2019: Highlights.

[2] Nikolaou A., Meric S., Fatta D. (2007). Occurrence patterns of pharmaceuticals in water and wastewater environments. Anal. Bioanal. Chem..

[3] Ebele A.J., Abou-Elwafa Abdallah M., Harrad S. (2017). Pharmaceuticals and personal care products (PPCPs) in the freshwater aquatic environment. Emerg. Contam. Handb..

[4] Miller T.H., Bury N.R., Owen S.F., MacRae J.I., Barron L.P. (2018). A review of the pharmaceutical exposome in aquatic fauna. Environ. Pollut..

[5] Wang J., Wang S. (2016). Removal of pharmaceuticals and personal care products (PPCPs) from wastewater: A review. J. Environ. Manag..

[6] Papageorgiou M., Kosma C., Lambropoulou D. (2016). Seasonal occurrence, removal, mass loading and environmental risk assessment of 55 pharmaceuticals and personal care products in a municipal wastewater treatment plant in Central Greece. Sci. Total Environ..

[7] Blair B., Nikolaus A., Hedman C., Klaper R., Grundl T. (2015). Evaluating the degradation, sorption, and negative mass balances of pharmaceuticals and personal care products during wastewater treatment. Chemosphere.

[8] Rocha M.J., Cruzeiro C., Ferreira C., Rocha E. (2012). Occurrence of endocrine disruptor compounds in the estuary of the Iberian Douro River and nearby Porto Coast (NW Portugal). Toxicol. Environ. Chem..

[9] Santos L.H.M.L.M., Gros M., Rodriguez-Mozaz S., Delerue-Matos C., Pena A., Barceló D., Montenegro M.C.B.S.M. (2013). Contribution of hospital effluents to the load of pharmaceuticals in urban wastewaters: Identification of ecologically relevant pharmaceuticals. Sci. Total Environ..

[10] Paíga P., Santos L.H.M.L.M., Ramos S., Jorge S., Silva J.G., Delerue-Matos C. (2016). Presence of pharmaceuticals in the Lis river (Portugal): Sources, fate and seasonal variation. Sci. Total Environ..

[11] Fernández-Rubio J., Rodríguez-Gil J.L., Postigo C., Mastroianni N., López de Alda M., Barceló D., Valcárcel Y. (2019). Psychoactive pharmaceuticals and illicit drugs in coastal waters of North-Western Spain: Environmental exposure and risk assessment. Chemosphere.

[12] Chen H., Liu S., Xu X.-R., Liu S.-S., Zhou G.-J., Sun K.-F., Zhao J.-L., Ying G.-G. (2015). Antibiotics in typical marine aquaculture farms surrounding Hailing Island, South China: Occurrence, bioaccumulation and human dietary exposure. Mar. Pollut. Bull..

[13] Kim H.-Y., Lee I.-S., Oh J.-E. (2017). Human and veterinary pharmaceuticals in the marine environment including fish farms in Korea. Sci. Total Environ..

[14] Kim K.-R., Owens G., Kwon S.-I., So K.-H., Lee D.-B., Ok Y.S. (2011). Occurrence and Environmental Fate of Veterinary Antibiotics in the Terrestrial Environment. Water Air Soil Pollut..

[15] Biel-Maeso M., Corada-Fernández C., Lara-Martín P.A. (2018). Monitoring the occurrence of pharmaceuticals in soils irrigated with reclaimed wastewater. Environ. Pollut..

[16] OSPAR Commission Hazardous Substances. https://www.ospar.org/work-areas/hasec/chemicals.

[17] Barra Caracciolo A., Topp E., Grenni P. (2015). Pharmaceuticals in the environment: Biodegradation and effects on natural microbial communities. A review. J. Pharm. Biomed. Anal..

[18] Bu Q., Shi X., Yu G., Huang J., Wang B. (2016). Assessing the persistence of pharmaceuticals in the aquatic environment: Challenges and needs. Emerg. Contam. Handb..

[19] (2018). Stockholm Convention on Persistent Organic Pollutants.

[20] Rodríguez-Mozaz S., Huerta B., Barceló D., Petrovic M.S., Elosegi A., Barceló D. (2015). Bioaccumulation of Emerging Contaminants in Aquatic Biota: Patterns of Pharmaceuticals in Mediterranean River Networks. Emerging Contaminants in River Ecosystems. The Handbook of Environmental Chemistry.

[21] Zenker A., Cicero M.R., Prestinaci F., Bottoni P., Carere M. (2014). Bioaccumulation and biomagnification potential of pharmaceuticals with a focus to the aquatic environment. J. Environ. Manag..

[22] De Solla S.R., Gilroy È.A.M., Klinck J.S., King L.E., McInnis R., Struger J., Backus S.M., Gillis P.L. (2016). Bioaccumulation of pharmaceuticals and personal care products in the unionid mussel Lasmigona costata in a river receiving wastewater effluent. Chemosphere.

[23] Howard P.H., Muir D.C.G. (2011). Identifying New Persistent and Bioaccumulative Organics Among Chemicals in Commerce II: Pharmaceuticals. Environ. Sci. Technol..

[24] Vandenberg L.N., Colborn T., Hayes T.B., Heindel J.J., Jacobs D.R., Lee D.-H., Shioda T., Soto A.M., vom Saal F.S., Welshons W.V. (2012). Hormones and Endocrine-Disrupting Chemicals: Low-Dose Effects and Nonmonotonic Dose Responses. Endocr. Rev..

[25] Contaminant Candidate List (CCL) and Regulatory Determination Chemical Contaminants—CCL 4. https://www.epa.gov/ccl/chemical-contaminants-ccl-4.

[26] Ji K., Liu X., Lee S., Kang S., Kho Y., Giesy J.P., Choi K. (2013). Effects of non-steroidal anti-inflammatory drugs on hormones and genes of the hypothalamic-pituitary-gonad axis, and reproduction of zebrafish. J. Hazard. Mater..

[27] Gonzalez-Rey M., Bebianno M.J. (2014). Effects of non-steroidal anti-inflammatory drug (NSAID) diclofenac exposure in mussel Mytilus galloprovincialis. Aquat. Toxicol..

[28] Gonzalez-Rey M., Bebianno M.J. (2013). Does selective serotonin reuptake inhibitor (SSRI) fluoxetine affects mussel Mytilus galloprovincialis?. Environ. Pollut..

[29] Marti E., Variatza E., Balcazar J.L. (2014). The role of aquatic ecosystems as reservoirs of antibiotic resistance. Trends Microbiol..

[30] Heys K.A., Shore R.F., Pereira M.G., Jones K.C., Martin F.L. (2016). Risk assessment of environmental mixture effects. RSC Adv..

[31] Watanabe H., Tamura I., Abe R., Takanobu H., Nakamura A., Suzuki T., Hirose A., Nishimura T., Tatarazako N. (2016). Chronic toxicity of an environmentally relevant mixture of pharmaceuticals to three aquatic organisms (alga, daphnid, and fish). Environ. Toxicol. Chem..

[32] Dietrich S., Ploessl F., Bracher F., Laforsch C. (2010). Single and combined toxicity of pharmaceuticals at environmentally relevant concentrations in Daphnia magna—A multigenerational study. Chemosphere.

[33] Petrovic M., Farré M., de Alda M.L., Perez S., Postigo C., Köck M., Radjenovic J., Gros M., Barcelo D. (2010). Recent trends in the liquid chromatography–mass spectrometry analysis of organic contaminants in environmental samples. J. Chromatogr. A.

[34] Larivière A., Lissalde S., Soubrand M., Casellas-Français M. (2017). Overview of Multiresidues Analytical Methods for the Quantitation of Pharmaceuticals in Environmental Solid Matrixes: Comparison of Analytical Development Strategy for Sewage Sludge, Manure, Soil, and Sediment Samples. Anal. Chem..

[35] Valera E., Babington R., Broto M., Petanas S., Galve R., Marco M.-P., Petrovic M., Barcelo D., Pérez S. (2013). Chapter 7—Application of Bioassays/Biosensors for the Analysis of Pharmaceuticals in Environmental Samples. Comprehensive Analytical Chemistry.

[36] Ejeian F., Etedali P., Mansouri-Tehrani H.-A., Soozanipour A., Low Z.-X., Asadnia M., Taheri-Kafrani A., Razmjou A. (2018). Biosensors for wastewater monitoring: A review. Biosens. Bioelectron..

[37] Suzuki H. (2000). Advances in the Microfabrication of Electrochemical Sensors and Systems. Electroanalysis.

[38] Zimmerman W.B. (2011). Electrochemical microfluidics. Chem. Eng. Sci..

[39] Lowe C.R. (1984). Biosensors. Trends Biotechnol..

[40] Fabry P., Siebert E., Gellings P.J., Bouwmeester H.J.M. (1997). Electrochemical sensors. the CRC Handbook of Solid State Electrochemistry.

[41] Dresselhaus M.S., Dresselhaus G., Eklund P.C. (1996). Science of Fullerenes and Carbon Nanotubes.

[42] Dai H., Wong E.W., Lieber C.M. (1996). Probing Electrical Transport in Nanomaterials: Conductivity of Individual Carbon Nanotubes. Science.

[43] Wu W., Krishnan S., Yamada T., Sun X., Wilhite P., Wu R., Li K., Yang C.Y. (2009). Contact resistance in carbon nanostructure via interconnects. Appl. Phys. Lett..

[44] Hanrahan G., Patil D.G., Wang J. (2004). Electrochemical sensors for environmental monitoring: Design, development and applications. J. Environ. Monit..

[45] Kurbanoglu S., Ozkan S.A. (2018). Electrochemical carbon based nanosensors: A promising tool in pharmaceutical and biomedical analysis. J. Pharm. Biomed. Anal..

[46] Oliveira T.M.B.F., Morais S. (2018). New Generation of Electrochemical Sensors Based on Multi-Walled Carbon Nanotubes. Appl. Sci..

[47] Feier B., Florea A., Cristea C., Săndulescu R. (2018). Electrochemical detection and removal of pharmaceuticals in waste waters. Curr. Opin. Electrochem..

[48] Fiel W., Borges P., Lins V., Faria R. (2019). Recent advances on the electrochemical transduction techniques for the biosensing of pharmaceuticals in aquatic environments. Int. J. Biosen. Bioelectron..

[49] Álvarez-Muñoz D., Rodríguez-Mozaz S., Maulvault A.L., Tediosi A., Fernández-Tejedor M., Van den Heuvel F., Kotterman M., Marques A., Barceló D. (2015). Occurrence of pharmaceuticals and endocrine disrupting compounds in macroalgaes, bivalves, and fish from coastal areas in Europe. Environ. Res..

[50] Moreno-González R., Rodríguez-Mozaz S., Huerta B., Barceló D., León V.M. (2016). Do pharmaceuticals bioaccumulate in marine molluscs and fish from a coastal lagoon?. Environ. Res..

[51] Núñez M., Borrull F., Pocurull E., Fontanals N. (2016). Pressurized liquid extraction followed by liquid chromatography with tandem mass spectrometry to determine pharmaceuticals in mussels. J. Sep. Sci..

[52] Valdés M.E., Huerta B., Wunderlin D.A., Bistoni M.A., Barceló D., Rodriguez-Mozaz S. (2016). Bioaccumulation and bioconcentration of carbamazepine and other pharmaceuticals in fish under field and controlled laboratory experiments. Evidences of carbamazepine metabolization by fish. Sci. Total Environ..

[53] Silva L.J.G., Pereira A.M.P.T., Rodrigues H., Meisel L.M., Lino C.M., Pena A. (2017). SSRIs antidepressants in marine mussels from Atlantic coastal areas and human risk assessment. Sci. Total Environ..

[54] Cunha S.C., Pena A., Fernandes J.O. (2017). Mussels as bioindicators of diclofenac contamination in coastal environments. Environ. Pollut..

[55] Álvarez-Muñoz D., Rodríguez-Mozaz S., Jacobs S., Serra-Compte A., Cáceres N., Sioen I., Verbeke W., Barbosa V., Ferrari F., Fernández-Tejedor M. (2018). Pharmaceuticals and endocrine disruptors in raw and cooked seafood from European market: Concentrations and human exposure levels. Environ. Int..

[56] Chiesa L.M., Nobile M., Malandra R., Panseri S., Arioli F. (2018). Occurrence of antibiotics in mussels and clams from various FAO areas. Food Chem..

[57] Kazakova J., Fernández-Torres R., Ramos-Payán M., Bello-López M.Á. (2018). Multiresidue determination of 21 pharmaceuticals in crayfish (Procambarus clarkii) using enzymatic microwave-assisted liquid extraction and ultrahigh-performance liquid chromatography-triple quadrupole mass spectrometry analysis. J. Pharm. Biomed. Anal..

[58] Kong C., Wang Y., Huang Y., Yu H. (2018). Multiclass screening of >200 pharmaceutical and other residues in aquatic foods by ultrahigh-performance liquid chromatography–quadrupole-Orbitrap mass spectrometry. Anal. Bioanal. Chem..

[59] Nödler K., Licha T., Bester K., Sauter M. (2010). Development of a multi-residue analytical method, based on liquid chromatography–tandem mass spectrometry, for the simultaneous determination of 46 micro-contaminants in aqueous samples. J. Chromatogr. A.

[60] Madureira T.V., Barreiro J.C., Rocha M.J., Rocha E., Cass Q.B., Tiritan M.E. (2010). Spatiotemporal distribution of pharmaceuticals in the Douro River estuary (Portugal). Sci. Total Environ..

[61] Lolić A., Paíga P., Santos L.H.M.L.M., Ramos S., Correia M., Delerue-Matos C. (2015). Assessment of non-steroidal anti-inflammatory and analgesic pharmaceuticals in seawaters of North of Portugal: Occurrence and environmental risk. Sci. Total Environ..

[62] Alygizakis N.A., Gago-Ferrero P., Borova V.L., Pavlidou A., Hatzianestis I., Thomaidis N.S. (2016). Occurrence and spatial distribution of 158 pharmaceuticals, drugs of abuse and related metabolites in offshore seawater. Sci. Total Environ..

[63] (2014). Medicine and Healthcare Products Statistics.

[64] The Top 300 of 2019. https://clincalc.com/DrugStats/Top300Drugs.aspx.

[65] Bollella P., Fusco G., Tortolini C., Sanzò G., Favero G., Gorton L., Antiochia R. (2017). Beyond graphene: Electrochemical sensors and biosensors for biomarkers detection. Biosens. Bioelectron..

[66] Saleh T.A., Fadillah G. (2019). Recent trends in the design of chemical sensors based on graphene–metal oxide nanocomposites for the analysis of toxic species and biomolecules. TrAC Trends Anal. Chem..

[67] Lima H.R.S., da Silva J.S., de Oliveira Farias E.A., Teixeira P.R.S., Eiras C., Nunes L., César C. (2018). Electrochemical sensors and biosensors for the analysis of antineoplastic drugs. Biosens. Bioelectron..

[68] Chen H., Bo L., He M., Mi G., Li J. (2015). Synthesis and Application of Graphene–Sodium Polyacrylate–Pd Nanocomposite in Amperometric Determination of Ciprofloxacin. Chem. Lett..

[69] Silva M., Cesarino I. (2019). Evaluation of a Nanocomposite Based on Reduced Graphene Oxide and Gold Nanoparticles as an Electrochemical Platform for Detection of Sulfamethazine. J. Compos. Sci..

[70] Chen B., Evans J.R.G., Greenwell H.C., Boulet P., Coveney P.V., Bowden A.A., Whiting A. (2008). A critical appraisal of polymer–clay nanocomposites. Chem. Soc. Rev..

[71] Work W., Horie K., Hess M., Stepto R. (2004). Definition of terms related to polymer blends, composites, and multiphase polymeric materials (IUPAC Recommendations 2004). Pure Appl. Chem..

[72] Chen C., Chen Y.-C., Hong Y.-T., Lee T.-W., Huang J.-F. (2018). Facile fabrication of ascorbic acid reduced graphene oxide-modified electrodes toward electroanalytical determination of sulfamethoxazole in aqueous environments. Chem. Eng. J..

[73] Lorenzetti A.S., Sierra T., Domini C.E., Lista A.G., Crevillen A.G., Escarpa A. (2019). Electrochemically Reduced Graphene Oxide-Based Screen-Printed Electrodes for Total Tetracycline Determination by Adsorptive Transfer Stripping Differential Pulse Voltammetry. Sensors.

[74] Sun X.-M., Ji Z., Xiong M.-X., Chen W. (2017). The Electrochemical Sensor for the Determination of Tetracycline Based on Graphene /L-Cysteine Composite Film. J. Electrochem. Soc..

[75] Liu W., Zhang J., Li C., Tang L., Zhang Z., Yang M. (2013). A novel composite film derived from cysteic acid and PDDA-functionalized graphene: Enhanced sensing material for electrochemical determination of metronidazole. Talanta.

[76] Li C., Zheng B., Zhang T., Zhao J., Gu Y., Yan X., Li Y., Liu W., Feng G., Zhang Z. (2016). Petal-like graphene–Ag composites with highly exposed active edge sites were designed and constructed for electrochemical determination of metronidazole. RSC Adv..

[77] Yang M., Guo M., Feng Y., Lei Y., Cao Y., Zhu D., Yu Y., Ding L. (2018). Sensitive Voltammetric Detection of Metronidazole Based on Three-Dimensional Graphene-Like Carbon Architecture/Polythionine Modified Glassy Carbon Electrode. J. Electrochem. Soc..

[78] Zheng B., Li C., Wang L., Li Y., Gu Y., Yan X., Zhang T., Zhang Z., Zhai S. (2016). Signal amplification biosensor based on DNA for ultrasensitive electrochemical determination of metronidazole. RSC Adv..

[79] Zhai H., Liang Z., Chen Z., Wang H., Liu Z., Su Z., Zhou Q. (2015). Simultaneous detection of metronidazole and chloramphenicol by differential pulse stripping voltammetry using a silver nanoparticles/sulfonate functionalized graphene modified glassy carbon electrode. Electrochim. Acta.

[80] Prado T.M.d., Cincotto F.H., Machado S.A.S. (2017). Spectroelectrochemical study of acetylsalicylic acid in neutral medium and its quantification in clinical and environmental samples. Electrochim. Acta.

[81] Dhanalakshmi N., Priya T., Thennarasu S., Sivanesan S., Thinakaran N. (2020). Synthesis and electrochemical properties of environmental free l-glutathione grafted graphene oxide/ZnO nanocomposite for highly selective piroxicam sensing. J. Pharm. Anal..

[82] Okoth O.K., Yan K., Liu L., Zhang J. (2016). Simultaneous Electrochemical Determination of Paracetamol and Diclofenac Based on Poly(diallyldimethylammonium chloride) Functionalized Graphene. Electroanalysis.

[83] Okoth O.K., Yan K., Feng J., Zhang J. (2018). Label-free photoelectrochemical aptasensing of diclofenac based on gold nanoparticles and graphene-doped CdS. Sens. Actuators B.

[84] Li Y., Zhao X., Li P., Huang Y., Wang J., Zhang J. (2015). Highly sensitive Fe3O4 nanobeads/graphene-based molecularly imprinted electrochemical sensor for 17β-estradiol in water. Anal. Chim. Acta.

[85] Moraes F.C., Rossi B., Donatoni M.C., de Oliveira K.T., Pereira E.C. (2015). Sensitive determination of 17β-estradiol in river water using a graphene based electrochemical sensor. Anal. Chim. Acta.

[86] Liu M., Ke H., Sun C., Wang G., Wang Y., Zhao G. (2019). A simple and highly selective electrochemical label-free aptasensor of 17β-estradiol based on signal amplification of bi-functional graphene. Talanta.

[87] Li J., Liu S., Yu J., Lian W., Cui M., Xu W., Huang J. (2013). Electrochemical immunosensor based on graphene–polyaniline composites and carboxylated graphene oxide for estradiol detection. Sens. Actuators B.

[88] Hu L., Cheng Q., Chen D., Ma M., Wu K. (2015). Liquid-phase exfoliated graphene as highly-sensitive sensor for simultaneous determination of endocrine disruptors: Diethylstilbestrol and estradiol. J Hazard. Mater..

[89] Gevaerd A., Banks C.E., Bergamini M.F., Marcolino-Junior L.H. (2019). Graphene Quantum Dots Modified Screen-printed Electrodes as Electroanalytical Sensing Platform for Diethylstilbestrol. Electroanalysis.

[90] Scala-Benuzzi M.L., Raba J., Soler-Illia G.J.A.A., Schneider R.J., Messina G.A. (2018). Novel Electrochemical Paper-Based Immunocapture Assay for the Quantitative Determination of Ethinylestradiol in Water Samples. Anal. Chem..

[91] Campuzano S., Yáñez-Sedeño P., Pingarrón J.M. (2019). Carbon Dots and Graphene Quantum Dots in Electrochemical Biosensing. Nanomaterials.

[92] Yu H., Zhang B., Bulin C., Li R., Xing R. (2016). High-efficient Synthesis of Graphene Oxide Based on Improved Hummers Method. Sci. Rep..

[93] Dresselhaus M.S., Dresselhaus G., Saito R. (1995). Physics of carbon nanotubes. Carbon.

[94] Thostenson E.T., Ren Z., Chou T.-W. (2001). Advances in the science and technology of carbon nanotubes and their composites: A review. Compos. Sci. Technol..

[95] Iijima S. (2002). Carbon nanotubes: Past, present, and future. Physica B.

[96] Britto P.J., Santhanam K.S.V., Rubio A., Alonso J.A., Ajayan P.M. (1999). Improved Charge Transfer at Carbon Nanotube Electrodes. Adv. Mater..

[97] Banks C.E., Davies T.J., Wildgoose G.G., Compton R.G. (2005). Electrocatalysis at graphite and carbon nanotube modified electrodes: Edge-plane sites and tube ends are the reactive sites. Chem. Commun..

[98] Banks C.E., Crossley A., Salter C., Wilkins S.J., Compton R.G. (2006). Carbon Nanotubes Contain Metal Impurities Which Are Responsible for the “Electrocatalysis” Seen at Some Nanotube-Modified Electrodes. Angew. Chem. Int. Ed..

[99] Rivas G.A., Rubianes M.D., Rodríguez M.C., Ferreyra N.F., Luque G.L., Pedano M.L., Miscoria S.A., Parrado C. (2007). Carbon nanotubes for electrochemical biosensing. Talanta.

[100] Agüí L., Yáñez-Sedeño P., Pingarrón J.M. (2008). Role of carbon nanotubes in electroanalytical chemistry: A review. Anal. Chim. Acta.

[101] Vashist S.K., Zheng D., Al-Rubeaan K., Luong J.H.T., Sheu F.-S. (2011). Advances in carbon nanotube based electrochemical sensors for bioanalytical applications. Biotechnol. Adv..

[102] Carneiro P., Morais S., Pereira M.C. (2019). Nanomaterials towards Biosensing of Alzheimer’s Disease Biomarkers. Nanomaterials.

[103] Gomes F.O., Maia L.B., Delerue-Matos C., Moura I., Moura J.J.G., Morais S. (2019). Third-generation electrochemical biosensor based on nitric oxide reductase immobilized in a multiwalled carbon nanotubes/1-n-butyl-3-methylimidazolium tetrafluoroborate nanocomposite for nitric oxide detection. Sens. Actuators B.

[104] Martínez N.A., Pereira S.V., Bertolino F.A., Schneider R.J., Messina G.A., Raba J. (2012). Electrochemical detection of a powerful estrogenic endocrine disruptor: Ethinylestradiol in water samples through bioseparation procedure. Anal. Chim. Acta.

[105] Nodehi M., Baghayeri M., Ansari R., Veisi H. (2020). Electrochemical quantification of 17α—Ethinylestradiol in biological samples using a Au/Fe3O4@TA/MWNT/GCE sensor. Mater. Chem. Phys..

[106] Liu X., Feng H., Liu X., Wong D.K.Y. (2011). Electrocatalytic detection of phenolic estrogenic compounds at NiTPPS|carbon nanotube composite electrodes. Anal. Chim. Acta.

[107] Biesaga M., Pyrzyńska K., Trojanowicz M. (2000). Porphyrins in analytical chemistry. A review. Talanta.

[108] Karla Lombello Coelho M., Nunes da Silva D., César Pereira A. (2019). Development of Electrochemical Sensor Based on Carbonaceal and Metal Phthalocyanines Materials for Determination of Ethinyl Estradiol. Chemosensors.

[109] Daneshvar L., Rounaghi G.H., Tarahomi S. (2016). Voltammetric paracetamol sensor using a gold electrode made from a digital versatile disc chip and modified with a hybrid material consisting of carbon nanotubes and copper nanoparticles. Microchim. Acta.

[110] Gorla F.A., Duarte E.H., Sartori E.R., Tarley C.R.T. (2016). Electrochemical study for the simultaneous determination of phenolic compounds and emerging pollutant using an electroanalytical sensing system based on carbon nanotubes/surfactant and multivariate approach in the optimization. Microchem. J..

[111] Shi P., Xue R., Wei Y., Lei X., Ai J., Wang T., Shi Z., Wang X., Wang Q., Mohammed Soliman F. (2020). Gold nanoparticles/tetraaminophenyl porphyrin functionalized multiwalled carbon nanotubes nanocomposites modified glassy carbon electrode for the simultaneous determination of p-acetaminophen and p-aminophenol. Arabian J. Chem..

[112] Gayen P., Chaplin B.P. (2016). Selective Electrochemical Detection of Ciprofloxacin with a Porous Nafion/Multiwalled Carbon Nanotube Composite Film Electrode. ACS Appl. Mater. Interfaces.

[113] Garrido J.M.P.J., Melle-Franco M., Strutyński K., Borges F., Brett C.M.A., Garrido E.M.P.J. (2017). β–Cyclodextrin carbon nanotube-enhanced sensor for ciprofloxacin detection. J. Environ. Sci. Health Part A.

[114] Zerkoune L., Angelova A., Lesieur S. (2014). Nano-Assemblies of Modified Cyclodextrins and Their Complexes with Guest Molecules: Incorporation in Nanostructured Membranes and Amphiphile Nanoarchitectonics Design. Nanomaterials.

[115] Arvand M., Gholizadeh T.M., Zanjanchi M.A. (2012). MWCNTs/Cu(OH)2 nanoparticles/IL nanocomposite modified glassy carbon electrode as a voltammetric sensor for determination of the non-steroidal anti-inflammatory drug diclofenac. Mater. Sci. Eng. C.

[116] Hu L., Zheng J., Zhao K., Deng A., Li J. (2018). An ultrasensitive electrochemiluminescent immunosensor based on graphene oxide coupled graphite-like carbon nitride and multiwalled carbon nanotubes-gold for the detection of diclofenac. Biosens. Bioelectron..

[117] Nasiri F., Rounaghi G.H., Ashraf N., Deiminiat B. (2019). A new electrochemical sensing platform for quantitative determination of diclofenac based on gold nanoparticles decorated multiwalled carbon nanotubes/graphene oxide nanocomposite film. Int. J. Environ. Anal. Chem..

[118] Roushani M., Shahdost-fard F. (2016). Covalent attachment of aptamer onto nanocomposite as a high performance electrochemical sensing platform: Fabrication of an ultra-sensitive ibuprofen electrochemical aptasensor. Mater. Sci. Eng. C.

[119] Iacob A., Manea F., Schoonman J., Vaszilcsin N. (2015). Voltammetric/amperometric detection of salicylic acid in water on carbon nanotubes-epoxy composite electrodes. WIT Trans. Built Environ..

[120] Vega D., Agüí L., González-Cortés A., Yáñez-Sedeño P., Pingarrón J.M. (2007). Voltammetry and amperometric detection of tetracyclines at multi-wall carbon nanotube modified electrodes. Anal. Bioanal. Chem..

[121] Nagles E., Alvarez P., Arancibia V., Baez M., Garreton V., Ehrenfeld N. (2012). Amperometric and voltammetric determination of oxytetracycline in trout salmonid muscle using multi-wall carbon nanotube, ionic liquid and gold nanoparticle film electrodes. Int. J. Electrochem. Sci.

[122] Yuan L., Jiang L., Hui T., Jie L., Bingbin X., Feng Y., Yingchun L. (2015). Fabrication of highly sensitive and selective electrochemical sensor by using optimized molecularly imprinted polymers on multi-walled carbon nanotubes for metronidazole measurement. Sens. Actuators B.

[123] Bueno A.M., Contento A.M., Ríos Á. (2013). Validation of a screening method for the rapid control of sulfonamide residues based on electrochemical detection using multiwalled carbon nanotubes-glassy carbon electrodes. Anal. Methods.

[124] Cesarino I., Cesarino V., Lanza M.R.V. (2013). Carbon nanotubes modified with antimony nanoparticles in a paraffin composite electrode: Simultaneous determination of sulfamethoxazole and trimethoprim. Sens. Actuators B.

[125] Yang M., Wu X., Hu X., Wang K., Zhang C., Gyimah E., Yakubu S., Zhang Z. (2019). Electrochemical immunosensor based on Ag+-dependent CTAB-AuNPs for ultrasensitive detection of sulfamethazine. Biosens. Bioelectron..

[126] Canevari T.C., Cincotto F.H., Nakamura M., Machado S.A.S., Toma H.E. (2016). Efficient electrochemical biosensors for ethynylestradiol based on the laccase enzyme supported on single walled carbon nanotubes decorated with nanocrystalline carbon quantum dots. Anal. Methods.

[127] Aragão J.S., Ribeiro F.W.P., Portela R.R., Santos V.N., Sousa C.P., Becker H., Correia A.N., de Lima-Neto P. (2017). Electrochemical determination diethylstilbestrol by a multi-walled carbon nanotube/cobalt phthalocyanine film electrode. Sens. Actuators B.

[128] Duan D., Si X., Ding Y., Li L., Ma G., Zhang L., Jian B. (2019). A novel molecularly imprinted electrochemical sensor based on double sensitization by MOF/CNTs and Prussian blue for detection of 17β-estradiol. Bioelectrochemistry.

[129] Veiga A., Dordio A., Carvalho A.J.P., Teixeira D.M., Teixeira J.G. (2010). Ultra-sensitive voltammetric sensor for trace analysis of carbamazepine. Anal. Chim. Acta.

[130] Deng K., Liu X., Li C., Hou Z., Huang H. (2017). An electrochemical omeprazole sensor based on shortened multi-walled carbon nanotubes-Fe3O4 nanoparticles and poly(2, 6-pyridinedicarboxylic acid). Sens. Actuators B.

[131] Chen L., Li K., Zhu H., Meng L., Chen J., Li M., Zhu Z. (2013). A chiral electrochemical sensor for propranolol based on multi-walled carbon nanotubes/ionic liquids nanocomposite. Talanta.

[132] Hemasa A.L., Naumovski N., Maher W.A., Ghanem A. (2017). Application of Carbon Nanotubes in Chiral and Achiral Separations of Pharmaceuticals, Biologics and Chemicals. Nanomaterials.

[133] Wang J., Musameh M., Lin Y. (2003). Solubilization of Carbon Nanotubes by Nafion toward the Preparation of Amperometric Biosensors. J. Am. Chem. Soc..

[134] Tkac J., Ruzgas T. (2006). Dispersion of single walled carbon nanotubes. Comparison of different dispersing strategies for preparation of modified electrodes toward hydrogen peroxide detection. Electrochem. Commun..

[135] Donnet J.-B., Bansal R.C., Wang M.-J. (1993). Carbon Black: Science and Technology.

[136] Sun J., Gan T., Meng W., Shi Z., Zhang Z., Liu Y. (2015). Determination of Oxytetracycline in Food Using a Disposable Montmorillonite and Acetylene Black Modified Microelectrode. Anal. Lett..

[137] Delgado K.P., Raymundo-Pereira P.A., Campos A.M., Oliveira O.N., Janegitz B.C. (2018). Ultralow Cost Electrochemical Sensor Made of Potato Starch and Carbon Black Nanoballs to Detect Tetracycline in Waters and Milk. Electroanalysis.

[138] Guaraldo T.T., Goulart L.A., Moraes F.C., Lanza M.R.V. (2019). Carbon black nanospheres modified with Cu (II)-phthalocyanine for electrochemical determination of Trimethoprim antibiotic. Appl. Surf. Sci..

[139] Deroco P.B., Rocha-Filho R.C., Fatibello-Filho O. (2018). A new and simple method for the simultaneous determination of amoxicillin and nimesulide using carbon black within a dihexadecylphosphate film as electrochemical sensor. Talanta.

[140] Deroco P.B., Fatibello-Filho O., Arduini F., Moscone D. (2019). Effect of Different Carbon Blacks on the Simultaneous Electroanalysis of Drugs as Water Contaminants Based on Screen-printed Sensors. Electroanalysis.

[141] Wong A., Santos A.M., Fatibello-Filho O. (2018). Simultaneous determination of paracetamol and levofloxacin using a glassy carbon electrode modified with carbon black, silver nanoparticles and PEDOT:PSS film. Sens. Actuators B.

[142] Machini W.B.S., Fernandes I.P.G., Oliveira-Brett A.M. (2019). Antidiabetic Drug Metformin Oxidation and in situ Interaction with dsDNA Using a dsDNA-electrochemical Biosensor. Electroanalysis.

[143] Qu K., Zhang X., Lv Z., Li M., Cui Z., Zhang Y., Chen B., Ma S., Kong Q. (2012). Simultaneous detection of diethylstilbestrol and malachite green using conductive carbon black paste electrode. Int. J. Electrochem. Sci.

[144] Pollap A., Knihnicki P., Kuśtrowski P., Kozak J., Gołda-Cępa M., Kotarba A., Kochana J. (2018). Sensitive Voltammetric Amoxicillin Sensor Based on TiO2 Sol Modified by CMK-3-type Mesoporous Carbon and Gold Ganoparticles. Electroanalysis.

[145] Kalate Bojdi M., Behbahani M., Mashhadizadeh M.H., Bagheri A., Hosseiny Davarani S.S., Farahani A. (2015). Mercapto-ordered carbohydrate-derived porous carbon electrode as a novel electrochemical sensor for simple and sensitive ultra-trace detection of omeprazole in biological samples. Mater. Sci. Eng. C.

[146] Ait Lahcen A., Ait Errayess S., Amine A. (2016). Voltammetric determination of sulfonamides using paste electrodes based on various carbon nanomaterials. Microchim. Acta.

[147] Dong X., He L., Liu Y., Piao Y. (2018). Preparation of highly conductive biochar nanoparticles for rapid and sensitive detection of 17β-estradiol in water. Electrochim. Acta.

[148] Santos A.M., Wong A., Vicentini F.C., Fatibello-Filho O. (2019). Simultaneous voltammetric sensing of levodopa, piroxicam, ofloxacin and methocarbamol using a carbon paste electrode modified with graphite oxide and β-cyclodextrin. Microchim. Acta.

[149] Motoc S., Manea F., Orha C., Pop A. (2019). Enhanced Electrochemical Response of Diclofenac at a Fullerene–Carbon Nanofiber Paste Electrode. Sensors.

[150] Eftekhari A., Fan Z. (2017). Ordered mesoporous carbon and its applications for electrochemical energy storage and conversion. Mater. Chem. Front..

[151] Serrano N., Castilla Ò., Ariño C., Diaz-Cruz M.S., Díaz-Cruz J.M. (2019). Commercial Screen-Printed Electrodes Based on Carbon Nanomaterials for a Fast and Cost-Effective Voltammetric Determination of Paracetamol, Ibuprofen and Caffeine in Water Samples. Sensors.

[152] Dhar D., Roy S., Nigam V.K., Kaur Brar S., Hegde K., Pachapur V.L. (2019). Chapter 10—Advances in protein/enzyme-based biosensors for the detection of pharmaceutical contaminants in the environment. Tools, Techniques and Protocols for Monitoring Environmental Contaminants.

[153] Liu J., Rinzler A.G., Dai H., Hafner J.H., Bradley R.K., Boul P.J., Lu A., Iverson T., Shelimov K., Huffman C.B. (1998). Fullerene Pipes. Science.

[154] Hu C., Ding Y., Ji Y., Xu J., Hu S. (2010). Fabrication of thin-film electrochemical sensors from single-walled carbon nanotubes by vacuum filtration. Carbon.

[155] Vallés C., David Núñez J., Benito A.M., Maser W.K. (2012). Flexible conductive graphene paper obtained by direct and gentle annealing of graphene oxide paper. Carbon.

[156] Martín A., Escarpa A. (2017). Tailor designed exclusive carbon nanomaterial electrodes for off-chip and on-chip electrochemical detection. Microchim. Acta.

[157] Torrinha Á., Montenegro M.C.B.S.M., Araújo A.N. (2019). Conjugation of glucose oxidase and bilirubin oxidase bioelectrodes as biofuel cell in a finger-powered microfluidic platform. Electrochim. Acta.

[158] Chen M., Qin X., Zeng G. (2017). Biodegradation of Carbon Nanotubes, Graphene, and Their Derivatives. Trends Biotechnol..

